# Protein complex prediction via dense subgraphs and false positive analysis

**DOI:** 10.1371/journal.pone.0183460

**Published:** 2017-09-22

**Authors:** Cecilia Hernandez, Carlos Mella, Gonzalo Navarro, Alvaro Olivera-Nappa, Jaime Araya

**Affiliations:** 1 Computer Science, University of Concepción, Concepción, Chile; 2 Center for Biotechnology and Bioengineering (CeBiB), Department of Computer Science, University of Chile, Santiago, Chile; 3 Center for Biotechnology and Bioengineering (CeBiB), Department of Chemical Engineering and Biotechnology, University of Chile, Santiago, Chile; University of Texas at San Antonio, UNITED STATES

## Abstract

Many proteins work together with others in groups called complexes in order to achieve a specific function. Discovering protein complexes is important for understanding biological processes and predict protein functions in living organisms. Large-scale and throughput techniques have made possible to compile protein-protein interaction networks (PPI networks), which have been used in several computational approaches for detecting protein complexes. Those predictions might guide future biologic experimental research. Some approaches are topology-based, where highly connected proteins are predicted to be complexes; some propose different clustering algorithms using partitioning, overlaps among clusters for networks modeled with unweighted or weighted graphs; and others use density of clusters and information based on protein functionality. However, some schemes still require much processing time or the quality of their results can be improved. Furthermore, most of the results obtained with computational tools are not accompanied by an analysis of false positives. We propose an effective and efficient mining algorithm for discovering highly connected subgraphs, which is our base for defining protein complexes. Our representation is based on transforming the PPI network into a directed acyclic graph that reduces the number of represented edges and the search space for discovering subgraphs. Our approach considers weighted and unweighted PPI networks. We compare our best alternative using PPI networks from *Saccharomyces cerevisiae* (yeast) and *Homo sapiens* (human) with state-of-the-art approaches in terms of clustering, biological metrics and execution times, as well as three gold standards for yeast and two for human. Furthermore, we analyze false positive predicted complexes searching the PDBe (Protein Data Bank in Europe) database in order to identify matching protein complexes that have been purified and structurally characterized. Our analysis shows that more than 50 yeast protein complexes and more than 300 human protein complexes found to be false positives according to our prediction method, i.e., not described in the gold standard complex databases, in fact contain protein complexes that have been characterized structurally and documented in PDBe. We also found that some of these protein complexes have recently been classified as part of a Periodic Table of Protein Complexes. The latest version of our software is publicly available at http://doi.org/10.6084/m9.figshare.5297314.v1.

## Introduction

Understanding biological processes at a cellular and system levels is an important task in all living organisms. Proteins are crucial components in many biological processes, such as metabolic and immune processes, transport, signaling, and enzymatic catalysis. Most proteins bind to other proteins in groups of interacting molecules, forming protein complexes to carry out biological functions. Berggård et al. [1] showed that more than 80% of proteins work in complexes. Moreover, many proteins are multifunctional, in the sense that they are part of different complexes according to the specific function required in the system. The discovery of protein complexes is of paramount relevance since it helps discover the structure-function relationships of protein-protein interaction networks (PPI networks), improving the understanding of the protein roles in different functions. Furthermore, understanding the roles of proteins in diverse complexes is important for many diseases, since biological research has shown that the deletion of some highly connected proteins in a network can have lethal effects on organisms [2].

Technological advances in biological experimental techniques have made possible the compilation of large-scale PPI networks for many organisms. Given the large volume of PPI networks, many mining algorithms have been proposed in recent years for discovering protein complexes. Research on PPI networks has shown that these networks have features similar to those of complex networks based on topological structures, such as small world [3] and scale free [4] properties. These networks are also formed by very cohesive structures [5]. These properties have been the inspiration for different computational approaches that identify protein complexes in PPI networks based on topological features. Most of these strategies model PPI networks as undirected graphs, where vertices represent proteins and edges are the interactions between them. Some strategies are based on density-based clustering [6, 7], community detection algorithms [8], dense subgraphs [9–11], and flow simulation-based clustering [12].

Since there are multifunctional proteins, some strategies also consider overlap among modules. Some strategies that are based on dense subgraphs use overlapping cliques, such as CFinder [10], distance metrics [9], and greedy algorithms for finding overlapping cohesive clusters [11] (ClusterONE). However, other methods do not consider overlapping structures, such as MCL [12] and the winner of the *Disease Module Identification DREAM Challenge* for subchallenge 1 (closed in November, 2016), which we call DSDCluster. DSDCluster is a method that first applies the DSD algorithm [13], which consists of computing a distance metric (Diffusion State Distance) for the connected genes in the network, and then applies spectral clustering. Other known algorithms for protein complex prediction are MCODE [14], RNSC [15], SPICI [16], DCAFP [17] and COREPEEL [18]. Complete surveys of computational approaches are available [19, 20].

An important characteristic of PPI networks is that they are noisy and incomplete, mainly due to the imprecisions of biological experimental techniques. To deal with this feature some researchers associate a weight to each edge representing the probability of the interaction being real [21–23]. Weights are inferred by analyzing primary affinity purification data of the biological experiments and defining scoring techniques for the protein interactions. These studies have motivated research on complex prediction tools that consider weights in the topological properties, including or not overlaps among complexes. Most of these computational strategies model PPI networks as undirected weighted graphs. Other approaches also include functional annotations of proteins to improve the quality of predicted complexes. Some of these techniques include functional annotation analysis as a pre-processing or post-processing step for predicted complexes [24, 25]; others include functional information in the complex prediction algorithms [7, 26]. Pre-processing strategies might also define weights in PPI networks based on functional similarity, and then use clustering algorithms on weighted graphs. In these approaches it is important both the definition of the similarity measure and the clustering algorithm, which should support overlap on weighted graphs. Post-processing strategies apply functional knowledge on predicted complexes, which is also biased by the quality of the predicted complexes. Applying functional annotations during the complex discovery is an interesting approach, but it is also biased to the quality of the functional similarity definition and the algorithm time complexity.

In order to validate predicted complexes, all computational strategies compare their results with gold standards used as references. Currently, CYC2008 [27] is the gold standard that reflects the current state of knowledge for yeast. This catalog contains 408 manually curated heteromeric protein complexes reliably supported by small-scale experiments reported in the literature. In fact CYC2008 was proposed as an update of MIPS (Munich Information Center of Protein Sequences) database [28], which was used as a reference until 2008. Another up-to-date reference for yeast is available at the SGD (Saccharomyces Genome Database) [29].

The prediction algorithms are important tools for updating the gold standards so that they reflect the latest biological knowledge. For example, one of the strategies used for building CYC2008 consisted in using the MCL (Markov Clustering) [12] algorithm for predicting protein complexes. This provided some complexes that were not in MIPS. Even though MCL is a very reliable algorithm, it does not support overlaps [19]. Using better prediction algorithms can therefore improve the current state of knowledge. Still, even though there are several prediction tools, there is no single method with dominating performance in terms of prediction quality and execution time for both small and large PPI networks.

### Our contribution

We propose an effective and efficient strategy for predicting protein complexes, using dense subgraphs built from complete bipartite graph patterns. Even though finding densely connected subgraphs is not a new idea and surely may not be the optimal property to look for in order to identify protein complexes (indeed, it is unknown which is that optimal property), this approach makes sense from different points of view.

First, it is biologically intuitive and evolutionarily logical to expect a low number of proteins to participate in many interactions, especially considering that such proteins should act as good control points for multiple related biological functions. This case is common in currently known biological networks and complexes and can explain why PPI networks have characteristics of “small-world” graphs. Second, analyzing the structural assembly of known complexes of more than two different proteins [30, 31], the majority of them implies highly connected protein nodes and cliques (see, for instance, all examples in Figure 3 of Marsh et al., 2015 [31], or Figure 6 in Ahnert et al., 2015 [30]), and there seems to be only a few ways in which protein complexes assemble. Third, protein complexes are thought to follow a few evolutionarily conserved ordered assembly pathways [32], which in the practice limits how many individual PPI interactions can be experimentally demonstrated for a given complex and how they can be translated into real complexes. In this scenario, looking for densely connected subgraphs in a PPI network may not be optimal, but it is a property representative of the new discoveries in complex assembly and it is efficient to at least screen and identify putative complexes. This has been demonstrated previously by the effective use of this approach in other algorithms, such as ClusterONE [11] and COREPEEL [18].

From an algorithmic point of view, our dense subgraph definition allows us to discover cliques and complete bipartite graphs that overlap. Since finding all maximal cliques in a graph is NP-complete [33], we propose a transformation of the input PPI network into an acyclic graph on which we design fast mining heuristics for finding dense subgraphs.

Our approach is somehow related to ClusterONE [11], in the sense that ClusterONE also uses a greedy heuristic that builds groups of vertices with high cohesiveness starting at seed vertices. In our approach, we first reduce the complexity of dense subgraph mining with the construction of the the acyclic graph from an input graph representing a PPI network. Then, we apply two different objective functions; the first enables the fast traversal of the acyclic graph and the second is used for detecting maximal dense subgraphs. COREPEEL, on the other hand, is related to our algorithm in the sense that it is also based on detecting dense subgraphs, but their approach uses core decomposition for finding quasi cliques in the graph (*core*) and then removes nodes with minimum degree (*peel*). Other approaches that also predict overlapping protein complexes are GMFTP [26] and DCAFP [17]. GMFTP builds an augmented network from a PPI network by adding functional information so that protein complexes can be discovered based on cliques identified from the augmented network. DCAFP also uses topological and functional information related to PPI networks.

We evaluate our algorithms using clustering and biological metrics on current yeast PPI networks, and compare our results with state-of-the-art strategies. We analyze the predicted complexes in terms of matching with three references for *Saccharomyces cerevisiae* (CYC2008, SGD, and MIPS) and two references for *Homo Sapiens* (PCDq [34], and CORUM [35]). We show that our approach improves upon the state of the art in quality and that it is fast in practice. DSDCluster achieves average performance (about the sixth best) in terms of clustering and biological metrics in all PPI networks, except on Biogrid-yeast where it is able to predict the greatest number of protein complexes that are in the CYC2008 gold standard (five more than the other methods). ClusterONE and COREPEEL provide good results and are also fast; however, our approach provides better results in terms of MMR, biological metrics and number of correct protein complexes based on gold standars in most of the PPI networks we analyzed in the manuscript. On the other hand, GMFTP and DCAFP provide good results but are several orders of magnitude slower than our approach.

As said, updating the gold standards is an important application of complex prediction tools. However, most prediction approaches do not discuss the predicted complexes that are false positives with respect to the current complexes in the references. These predicted complexes are not necessarily incorrect results; they can actually be new complexes that have not yet been discovered, or can be part of biological evidence not captured in the construction of the current gold standards.

In our work, we analyze the false-positive protein complexes predicted by our method (i.e., complexes not described in the gold standards), and report on our findings. Precisely, we searched for false-positive complexes that had been purified and structurally characterized in the PDBe (Protein Data Bank in Europe) database.

Our results show that we achieve good performance in discovering protein complexes, while obtaining results of good quality. Compared with the state of the art, we are the first or the second best method considering the MMR measure [11] in both small and large PPI networks. Further, our automatic false positive analysis shows that many of our false positives in fact contain small curated protein complexes that are reported in PDBe and not found in gold standards: more than 50 on yeast and 300 on human proteins.

## Materials and methods

In this section we present our graph definitions for modeling PPI networks, formulate the problem of finding dense subgraphs, and describe the algorithms for detecting dense subgraphs. Our approach enables us to find dense subgraphs that usually overlap among them. We then describe different alternatives for mapping dense subgraphs to protein complexes.

### Graph models for PPI networks

Since the interactions among proteins in a PPI are symmetric, these networks are usually modeled as undirected graphs, where proteins are vertices and interactions between proteins are edges. We represent a PPI network with adjacency lists, where each adjacency list contains the set of neighbors of a protein. In order to find complexes, we represent each undirected edge {*u*, *v*} as two directed edges (*u*, *v*) and (*v*, *u*). Therefore, *u* appears in the adjacency list of *v* and *v* appears in the adjacency list of *u*. The PPI network is then modeled as a directed graph *G* = (*V*, *E*, *w*), where *V* is the set of vertices (proteins), *E* ⊆ *V* × *V* is the set of edges (protein-protein interactions), and *w*: *E* → [0, 1] is a function that maps an edge to a real number between 0 and 1 that represents the probability that an interaction is real.

### Preliminaries

We first represent a protein-protein interaction network as a graph, where the protein names of the network are represented as vertices in the graph with numeric ids. Thus, each protein name must be mapped to a unique numeric id. Mapping protein names to numeric ids can be done using any *Node ordering algorithm*, such as random, lexicographic, by degree, BFS traversal, and DFS traversal, among others.

Our algorithm for finding dense subgraphs looks for cliques and complete bipartite subgraphs in the PPI network. The process of finding good dense subgraphs is run over an acyclic graph called *DAPG*, which is built from the input PPI network.

**Definition 1** Directed Acyclic Prefix Graph (DAPG)

*Given a graph G* = (*V*, *E*), *a set V*′ ⊆ *V and a total order ϕ* ⊆ *V* × *V*, *we define a directed acyclic graph DAPG* = (*N*, *A*), *as follows*:

- *N* = ⋃_*v*′ ∈ *V*′_ *adjlist*_*ϕ*_(*v*′),
- *A* = {(*u*_1_, *u*_2_) ∈ *N* × *N*, ∃*v*′ ∈ *V*′, *u*_1_ *and u*_2_ *are consecutive in adjlist*_*ϕ*_(*v*′)},

where *adjlist*_*ϕ*_(*v*) = 〈*u* ∈ *V*, (*v*, *u*) ∈ *E*〉 is the adjacency list of node *v* in *G* = (*V*, *E*), listed in the total order *ϕ*.

**Fig 1:**
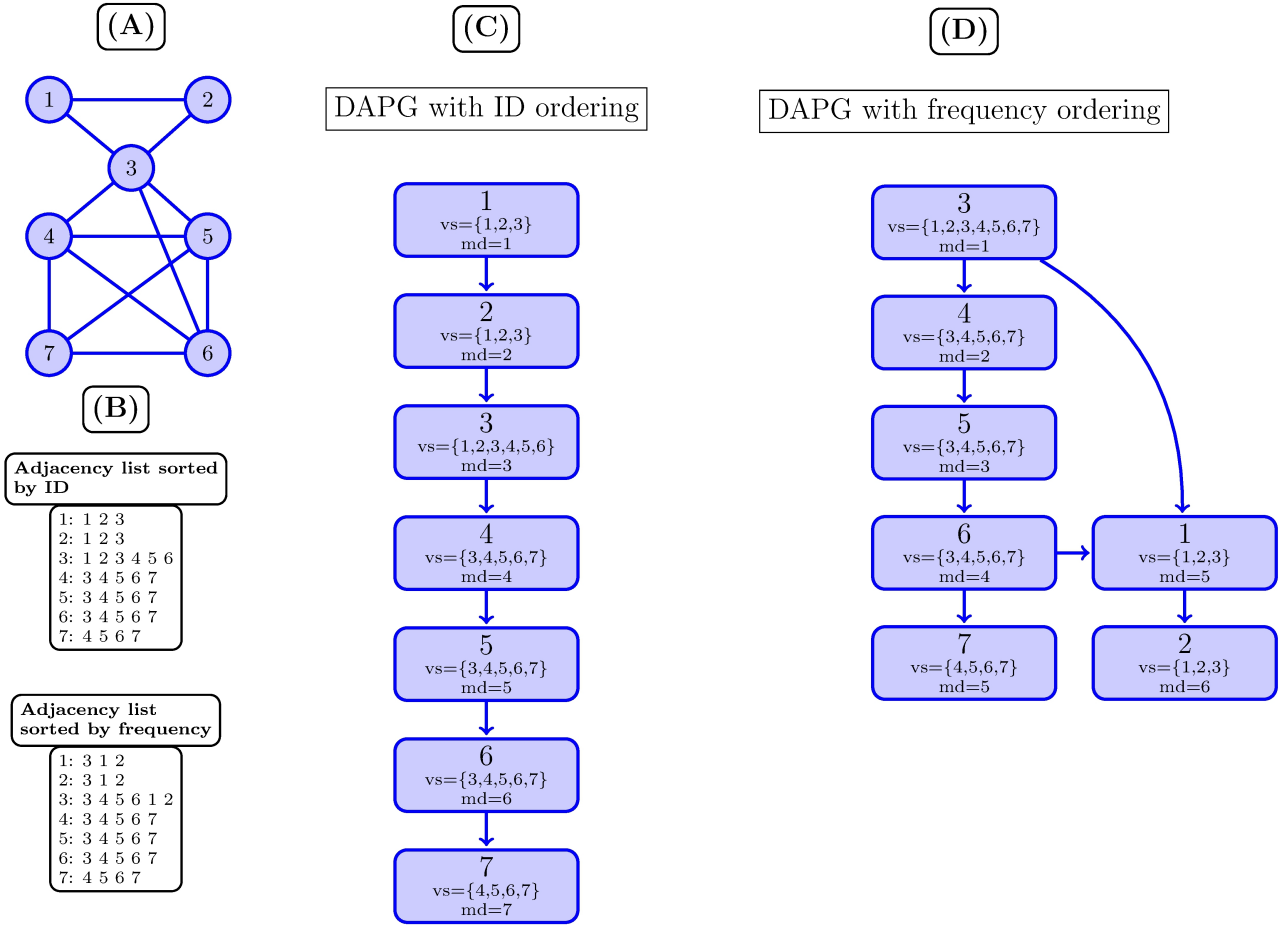
DAPG example. (A) shows a PPI as an undirected graph. (B) shows a PPI network as an adjacency list. (C) shows the DAPG using total order function *ϕ* (ID) and (D) shows the DAPG using total order function *ϕ* FREQUENCY.

Using a total order *ϕ* for the adjacency lists of *G* ensures that DAPG has no cycles. We consider two possible total orders *ϕ*: *ID* sorts the nodes by their ids, whereas *FREQUENCY* sorts them by their indegree, or number of times they appear in all the adjacency lists of *V*′. Fig 1 shows the use of both relations.

We say that a node *u*′ is the *parent* of *u* in DAPG iff (*u*′, *u*) ∈ *A*, and call *root* a node with no parents. A *path* is a sequence of nodes in DAPG, (*u*_*i*_, *u*_*i*+1_) ∈ *A*, with *i* = 1, …, *n* − 1.

In addition, we define attributes for any node *u* ∈ *N* in DAPG based on the input graph *G* = (*V*, *E*), as follows:

- *label*: a unique identifier given to a node *v* ∈ *V* in *G*.
- *vertexSet*(*u*) = {*v* ∈ *V*′, (*v*, *u*) ∈ *E*}.

In words, the *vertexSet* of a node *u* ∈ *N* is the set of vertices *v* ∈ *V*′ pointing to *u*, that is, whose adjacency lists *adjlist*(*v*) contain *u*. Note that the FREQUENCY order sorts nodes *u* by |*vertexSet*(*u*)|.

Let us now define the types of dense subgraphs we will detect.

**Definition 2** Dense subgraph (DSG)

*A dense subgraph DSG*(*S*, *C*) *of G* = (*V*, *E*) *is any graph G*′(*S* ∪ *C*, *S* × *C*), *where S*, *C* ⊆ *V*, *and S* × *C* ⊆ *E*, *that is*, *it contains all the edges from a subset of nodes S to another subset C*. *Our implementation removes possible self-loops*.

Note that Definition 2 includes cliques (*S* = *C*) and bicliques (*S* ∩ *C* = ∅, known as complete bipartite graphs), but also more general subgraphs where *S* ∩ *C* ≠ 0.

The following lemma defines the way we will find dense subgraphs.

**Lemma** *Given a DAPG D* = (*N*, *A*), *a path P* = (*u*_1_, *u*_2_, …, *u*_*h*_) *in D*, *and a set R* ⊆ *P*, *a valid dense subgraph DSG* = (*S*, *C*) *is defined as S* = ⋂_*u*∈*R*_ *vertexSet*(*u*) *and C* = *R*.

In order to find a promising path in DAPG starting from a given node *u*, we define an *inverse traveler function*, as follows.

**Definition 3** Inverse traveler function

*An inverse traveler in DAPG is a partial function t*: *N* → *N*, *such that t*(*u*) *is a parent of u in DAPG*. *It gives no answer only when u is a root in N*.

An inverse traveler function traverses a set of nodes in DAPG, moving from a node to one of its parents, up to a root. Therefore, given a node *u*, the nodes in the path *P*_*u*_ are be determined by applying the function *t* repeatedly on *u*: *u* → *t*(*u*) → (*t* ∘ *t*)(*u*) → … → *root*.

Once we have a path *P*_*u*_ we determine a set *R*_*u*_ ⊆ *P*_*u*_, with *u* ∈ *R*_*u*_, that maximizes a given objective function *f*_*obj*_ defined as follows.

**Definition 4** *An objective function is a function* $f_{obj}:H\to N_{0}$, *where* H *is the universe of dense subgraphs of the form H* = (*S*, *C*) *based on Definition 2*.

Objective functions maximize some feature of dense subgraphs, aiming at detecting good ones. The functions used in this work are based on the number of edges in the dense subgraphs, or on a weighted density measure. They are listed in Table 1.

**Table 1:** Inverse traveler and objective functions.

**Inverse traveler functions**	
Deepest	*u* ↦ *parent* *p*, *with maximum* *maxDepth*(*p*) = *maxDepth*(*u*) − 1
Sharing	*u* ↦ *parent* *p*, *with maximum* |*u*.*vertexSet* ∩ *p*.*vertexSet*|
**Objective functions**	
UNONE	Intersection size: *f*_*obj*_(*dsg*) = |*S* ∩ *C*|.
WDEGREE	Weighted degree density: $f_{obj}\left(dsg\right)=\frac{\sum_{a\in E(S\times C)}w\left(a\right)}{\left|S\cup C\right|}$ where *W*(*a*) is the weight value in the edge *a*.
WEDGE	Weighted edge density: $f_{obj}\left(dsg\right)=\frac{2\times \sum_{a\in E(S\times C)}w\left(a\right)}{\left|S\cup C|\times (|S\cup C|-1\right)}$
FWEDGREE	Full Weighted degree density: WDEGREE of the induced subgraph of *S* ∪ *C*.
FWEDGE	Full Weighted degree density: WEDGE of the induced subgraph of *S* ∪ *C*.

An important advantage of our approach is that it enables the easy extension of new traveler and objective functions. New traveler functions might improve the mining process for discovering dense subgraghs and new objective functions might include biological knowledge to discover subgraphs with biological significance.

Our problem can then be formulated as follows.

**Problem**: Detecting Maximal Dense Subgraphs

*For a given graph G* = (*V*, *E*, *w*), *represented by a DAPG* (*N*, *A*), *a weight function w*: *E* → [0, 1], *a traveler function t*, *and a given objective function f*_*obj*_, *output a set of maximal dense subgraphs* (*S*, *C*) *of G*.

### Algorithms

Our algorithm first represents a PPI network as a graph *G* where each protein in the network is a vertex with a numeric id. Mapping protein names to numeric ids can be performed using any node ordering algorithm. In this work, we use six different mappings. *First* maps protein names to numeric ids in the order in which proteins are read from the PPI network. *Lexicographic* sorts the protein names and then assigns the numeric ids in that order. *Degree* sorts the proteins by decreasing degree in the network and then assigns the numeric ids in that order. *Random* maps protein names to numeric ids randomly. Finally, *BFS* and *DFS* map proteins names based on the breadth-first or depth-first search network traversal, respectively.

The algorithm we propose for discovering dense subgraphs proceeds in two phases. The first phase builds an acyclic graph DAPG from *G*, using a total ordering function in the adjacency lists. As mentioned, we propose two total ordering functions: ID and FREQUENCY. The second phase consists in discovering dense subgraphs based on optimizing two objective functions: one guides the traversal on DAPG and the other specifies which nodes to choose.

Lemma 1 enables the detection of dense subgraphs from DAPG, however, even for a given path *P*, finding all the possible sets *R* in the path requires time exponential in the number of nodes in the path. Finding the best paths *P* in DAPG is also exponential-time. Instead, we design an efficient mining heuristic for discovering dense subgraphs in DAPG.

The main mining heuristic is based on finding at most one dense subgraph starting at each node in DAPG. This approach enables us to find dense subgraphs that might overlap. The heuristic is based on finding a promising path *P*_*u*_ = (*u*_1_, *u*_2_, …*u*_*n*_) so that *u*_1_ is a root in DAPG. We find a promising path in DAPG starting from a given node *u* using an *inverse traveler function* given in Definition 3.

The core of our mining technique starts at each node *v* in DAPG and walks its way to the previous node in the path up to a root. Along the path, we maintain in set *S* the intersection of the *vertexSet* of the nodes in a subset of the visited nodes (those which provide a better partial *DSG*), while we maintain in set *C* the *labels* of the nodes of the selected subset. Note that, at each point, (*S* ∪ *C*, *S* × *C*) is indeed a valid graph. From all those DSGs, we retain only the “best one”. We determine the “best DSG” using and objective function (*f*_*obj*_), which is a configuration parameter.

We can customize the core of the mining technique based on an *inverse traveler function*, *t*, to obtain a promising path *P* in DAPG, and an *objective function*, *f*_*obj*_, to discover dense subgraphs given by Definition 2. This approach is flexible to favor given features of dense subgraphs, and allows the exploration of different ideas for determining alternative paths to improve the quality of the results.

We consider the *inverse traveler* and *objective functions* defined in Table 1.

In order to efficiently implement the inverse traveler function *Deepest* in Table 1, we attach another attribute to each node in DAPG, called *maxDepth*, which corresponds to the length of the longest path from a root to each node and it is defined as follows.

**Definition 5** MaxDepth

*Given a dag DAPG* = (*N*, *A*), *then* ∀*u* ∈ *N*:

$$\begin{array}{c} maxDepth\left(u\right)=\{\begin{array}{cc} 1 & if\;u\;is\;root\\ max_{(p,u)\in A}\left(maxDepth\left(p\right)\right)+1, & otherwise \end{array} \end{array}$$

Finally, the algorithm returns the best DSG it could find starting from node *v*.

We run the algorithm starting at each node *u* in DAPG, so one DSG is obtained per starting node *u*. We only collect the maximal DSGs among those (i.e., DSGs that are not subsets of others).

All algorithms are presented in S1 File.

Fig 1 shows an example of a PPI network represented with a DAPG using the inverse traveler function *Deepest*, *f*_*obj*_ = *UNONE*, using total order functions *ϕ* sorting by ID (C) and by FREQUENCY (D). With this representation, we are able to discover cliques *C*1 = (1, 2, 3), *C*2 = (3, 4, 5, 6) and *C*3 = (4, 5, 6, 7).

### Analysis of the algorithms

Let *n* be the number of nodes in DAPG, *h* ≤ *n* be the longest path, and *e* ≤ *n* be the maximum number of neighbors of a node. Then, our algorithm starts from each node in DAPG, with an initial *vertexSet* of size at most *e*, and walks some path upwards to the root, performing at most *h* steps. At each step it must compute the distance traveler function, which in our examples costs *O*(1) or *O*(*e*) time. It also intersects the *vertexSet* of the new node with the current candidates, in time *O*(*e*), and determines whether or not to keep the current node in the set *C*. All the criteria we use for the latter can be computed in time *O*(*e*). Therefore, the total time of this process is *O*(*nhe*).

Let *m* be the maximum number of maximal subgraphs produced along the process. Once the new subgraph is produced, we compare it with the *O*(*m*) current maximal subgraphs, looking for those that include or are included in the new one, in order to remove the included ones (or the new one). This costs *O*(*nme*) time.

The total cost is therefore *O*(*ne*(*h* + *m*)). This is *O*(*n*^3^) in the worst case, but much less in practice. For example, in Collins we have *n* = 1,622, *e* = 127, *h* = 187, and *m* = 12, and therefore *ne*(*h* + *m*) is 25,273*n*, which is 100 times less than *n*^3^ = 2,630,884*n*

### Protein complex prediction

We define protein complexes from the DSGs we discover in PPI networks. Since we obtain at most one DSG starting at each node in DAPG, our algorithm is able to obtain DSGs that are in overlap. Let a parameter *minSize* define the minimum size of a candidate complex. Then, each DSG(*S*, *C*) is considered as a candidate complex with nodes *S* ∪ *C* whenever |*S* ∪ *C*| ≥ *minSize*.

We generate predicted complexes from candidate complexes based on two different filter options: NONE, where a predicted complex is always a candidate complex, and UNION, where a predicted complex is formed by the set union of the complex pairs with overlap score (Eq 1) greater than a *threshold* (we used *threshold* = 0.8).

## Experimental setup

We implemented the algorithms in C++ and executed all the experiments on a 64-bit Linux machine with 8GB of main memory and with an Intel CPU with i7 2.7GHz. All state-of-the-art methods are also executed on the same machine, except COREPEEL, which provide its method through its web site.

We used yeast (Saccharomyces cerevisiae) and human (Homo Sapiens) PPI networks for experimental evaluation. Specifically, we used the following yeast PPI networks: Collins [21], Krogan core and Krogan extended [22], Gavin [23], DIP-yeast (available in [18]) and BioGrid (version 3.4.138) for yeast (available at http://thebiogrid.org). We used human PPI networks Biogrid (version 3.4.138) and HPRD [36]. We compared our complex prediction results against the up-to-date complex yeast reference CYC2008 [27], SGD (available at http://www.yeastgenome.org), and MIPS (obtained from the ClusterONE distribution [11]). For human proteins we used PCDq [34] and CORUM [35]. Table 2 shows the main statistics of PPI networks we used and Table 3 displays the number of complexes of each reference plus the number of complexes obtained by merging them. Since performing an exact merging of gold standards might be difficult, we approximate the merge procedure as follows: If the same protein complex name is found, then the merged version contains only one copy. If the protein complex names are different and the complexes contain the same proteins, then the merged version also contains one copy. If both the complex name and the proteins are different, then the merged reference contains both complexes.

**Table 2:** Main statistics of PPI networks.

	Proteins	Interactions	Avg degree
Saccharomyces cerevisiae (yeast)			
Collins	1,622	9,074	5.59
Krogan core	2,708	7,123	2.63
Krogan extended	3,672	14,317	3.89
Gavin	1,855	7,669	4.13
DIP-yeast	4,638	21,377	4.60
Biogrid yeast	6,436	229,409	35.64
Homo sapiens (human)			
HPRD	9,453	36,867	3.90
Biogrid human	17,545	233,688	13.31

**Table 3:** Main statistics of protein complex references.

Name	Complexes	URL
Saccharomyces cerevisiae		
CYC2008	408	http://wodaklab.org/CYC2008/
SGD	372	http://www.yeastgenome.org/download-data/curation
MIPS	203	http://www.paccanarolab.org/clusterone/
CYC2008, SGD	582	Built
CYC2008, SGD, MIPS	614	Built
Homo sapiens		
CORUM	1,679	http://mips.helmholtz-muenchen.de/genre/proj/corum/
PCDq	1,263	http://h-invitational.jp/hinv/pcdq/
CORUM, PCDq	2,881	Built

For biological metrics, we also used current state-of-the-art gene ontology and annotations, available at http://www.geneontology.org.

We considered state-of-the-art complex prediction methods such as ClusterONE [11], MCL [12], CFinder [10], GMFTP [26], MCODE [14], RNSC [15], SPICI [16], DCAFP [17] and COREPEEL [18]. For each method we used the parameters that provided the best results.

To evaluate the effectiveness of our clustering approach we considered clustering and biological metrics. Clustering metrics measure the quality of the complexes in terms of how well the predicted complexes are related to the reference complexes. Biological metrics assess the probability that proteins in predicted complexes form real complexes (given by a reference) based on the relationship among the proteins in terms of their localization and the annotations.

Proposed methods usually measure the degree of matching between a predicted and a real complex [19]. This metric is usually called Overlap Score (OS) or Network Affinity (NA). If *pc* is the set of vertices forming a predicted complex and *rc* the set of vertices forming a complex in the reference, we have Eq 1 for OS:

$$\begin{array}{c} OS\left(pc,rc\right)=\frac{{\left|pc\cap rc\right|}^{2}}{\left|pc|\times |rc\right|} \end{array}$$ (1)

Many research works declare a match between a predicted and a reference complex when *OS* ≥ *w* (generally *w* = 0.2 or 0.25 [19]).

We used three clustering evaluation metrics usually found in complex prediction evaluations: FMeasure, Accuracy (Acc) and Maximum Matching Ratio (MMR).

FMeasure is defined in terms of Precision and Recall, which depend on the definition of True Positives (TP), False Positives (FP) and False Negatives (FN). TP is the number of predicted complexes with an OS over a threshold value for some reference complex, and FP is the total number of predicted complexes minus TP. FN is the number of complexes known in the reference that are not matched by any predicted complex. Precision and Recall are metrics that measure, respectively, how many predicted complexes are correct with respect to the total number of predicted complexes, and how many reference complexes are correctly predicted. Eq 2 gives their formulas. It also gives the formula for FMeasure, which is the harmonic mean of Precision and Recall and is used, among other metrics, to measure the overall performance of clustering algorithms.

$$\begin{array}{c} \begin{array}{cc} Precision & =\frac{TP}{TP+FP}\\ Recall & =\frac{TP}{TP+FN}\\ FMeasure & =\frac{2\times Recall\times Precision}{Recall+Precision} \end{array} \end{array}$$ (2)

*Acc* is the geometric mean of Sensitivity *S*_*n*_ and Positive Predicted Value *PPV*. *S*_*n*_ shows how good is the identification of proteins in the reference complexes in terms of coverage, and *PPV* indicates the probability of that the predicted complexes are TP. Eq 3 displays the equations for *S*_*n*_, *PPV*, and *Acc*. *T*_*ij*_ is the number of proteins in common between the *i*_*th*_ reference complex and *j*_*th*_ predicted complex; *n* is the number of complexes in the reference and *m* the number of predicted complexes; *N*_*i*_ is the number of proteins in the *i*_*th*_ reference complex, and $T_{j}=\sum_{i=1}^{n}T_{ij}$.

$$\begin{array}{c} \begin{array}{cc} S_{n} & =\frac{\sum_{i=1}^{n}max_{j}\left\{T_{ij}\right\}}{\sum_{i=1}^{n}N_{i}}\\ PPV & =\frac{\sum_{j=1}^{m}max_{i}\left\{T_{ij}\right\}}{\sum_{j=1}^{m}N_{j}}\\ Acc & ={\left(S_{n}\times PPV\right)}^{1/2} \end{array} \end{array}$$ (3)

Since several research works use FMeasure and Acc as clustering evaluation metrics, we included them as well. However, they are not free of problems. For instance, Acc penalizes predicted complexes that do not match any of the reference complexes, when some of the predicted complexes might indeed be undiscovered complexes.

We also used MMR measure, introduced by Nepusz et al. [11] to avoid the penalization of accuracy metrics over clusters with significant overlaps. MMR is based on a maximal one-to-one mapping between predicted and reference complexes. MMR represents a bipartite graph where one set of nodes is formed by the predicted complexes and the other by the reference complexes. Each edge has a weight representing the overlap score between the two vertices. The maximum weighted bipartite matching on this graph measures the quality of predicted complexes with respect to the reference complexes. The MMR score is given by the sum of the weights of the edges on this graph divided by the number of reference complexes. MMR offers a good comparison between predicted and reference complexes, penalizing those cases when reference complexes are found in two predicted complexes with high overlap.

In order to compute the MMR (Eq 4), ClusterONE first matches each reference complex (*rc*_*i*_) to a predicted complex (*pc*_*j*_) that maximizes the average *OS* over all reference complexes (considering a minimum *OS* ≥ 0.2).

$$\begin{array}{c} MMR=\frac{\sum_{i=1}^{\left|RC\right|}OS\left(rc_{i},pc_{j}\right)}{\left|RC\right|} \end{array}$$ (4)

One important feature of PPI networks is that they are incomplete and noisy. Biological processes for discovering protein interactions are not error free. In consequence, PPI networks might miss proteins with their interactions or include interactions that are not real. Algorithms should consider this feature to improve mining results [19]. This fact can be observed by looking at the proteins that are in PPI networks and the proteins that are in the reference. Nepusz et al. [11] consider the three following cases for proteins in PPI networks and the reference.

1. Proteins appearing in the PPI and in the reference.
2. Proteins appearing in the PPI, but not in the reference.
3. Proteins appearing in the reference, but not in the PPI.

Evaluating mining algorithms for the cases (1) and (2) is straightforward since protein interaction can be captured by the mining algorithm. Complexes found in case (2) might owe to mistakes on the mining algorithm or incompleteness of the reference, therefore this last case might require an analysis of the false positives generated by the mining algorithm. However, finding complexes in case (3) is impossible for any mining algorithm based on clustering. A possible simple solution to evaluate a mining algorithm would be not to consider reference complexes containing proteins unknown in the PPI, but if these protein interactions are missing in large predicted complexes then there might not be a good reason to eliminate the complete complex. Based on these considerations Nepusz et al. [11] propose filtering the references for evaluating a mining algorithm. The procedure is given as follows:

1. Identify all proteins that had at least one known interaction with other proteins in the input PPI.
2. For each complex in the reference, identify its proteins and compute the set intersection with all proteins in the input PPI.
3. If the set intersection size of a reference complex in the previous step is less than half of the size of the complete reference complex, such reference complex is eliminated because too many proteins are missing in the input PPI, and even if this complex is predicted might not be because of the quality of the algorithm.
4. If the set intersection size of a reference complex is greater than half of the size of the complete reference complex, the reference complex is considered but all proteins that are unknown to the PPI are eliminated. This action does not improve the quality of the mining algorithm since all algorithms are assessed on the same reference and those proteins could not be inferred anyways.

In order to provide a fair way to compare our approach against other proposed methods, we used the implementation just described [11], available at https://github.com/jboscolo/RH/find/master. Such implementation includes the computation of FMeasure, Acc and MMR.

### Biological measures

Besides clustering measures, we consider biological relevance metrics. In this context we used *Colocalization* and *Gene Ontology Similarity (GoSim)*. *Colocalization* measures the relationship of proteins based on where they are located in the cell and organism. The idea is that since protein complexes are assembled to perform a specific function, proteins within the same complex tend to be close to each other [37]. The idea of *GoSim* comes from the Gene Ontology Annotations, which basically describe the functions in which proteins work. Since protein complexes are formed to perform on specific functions, proteins forming a complex tend to share similar functionality [38]. We used the software ProCope to measure *Colocalization* and *GoSim*. ProCope is available at https://www.bio.ifi.lmu.de/software/procope/index.html [39].

We also include a biological measure that measures the biological significance of predicted protein complexes using enrichment analysis. In order to compute the biological significance of predicted complexes we use the same method described in [40], taking into account the p-values of predicted complexes, which represent the probability of co-occurrence of protein with common functions. As in [40], we also used BINGO [41], which is a Cytoscape [42] plugin that computes which GO categories are statistically overrepresentated using hypergeometric test in a set of genes. A low p-value for a set of genes in a predicted complex indicates that those proteins are statistically relevant in the complex. Typically considering a p-value < 0.01 is considered as a significant predicted complex. We measure significant complexes as percentage (SC).

### Clustering performance results

As mentioned in previous sections, we considered clustering metrics used by other clustering strategies such as FMeasure, Accuracy (Acc) and Maximum Matching Ratio (MMR). Specifically, we used the ClusterONE implementation of Acc and MMR metrics and we added support for FMeasure to compare all clustering techniques considered for comparison. ClusterONE implementation eliminates reference complexes that contain more than 50% of proteins that are unknown (i.e., proteins that are absent in the PPI network) and removes unknown proteins of complexes that contain less than 50% of such proteins.

### Parameter tuning

First, we define different node ordering algorithms to map the protein names to unique numeric ids in the graph. We consider the node ordering algorithms already described: *First*, *Random*, *Degree*, *Lexicographic*, *BFS*, and *DFS*.

We compared our results according to the different parameters we have in our algorithms. We present a summary of the main parameters we provide in our approach in Table 4. With *Protein Mapping* we specify the text file describing the mapping from proteins to numeric ids. With *Graph Type* we specify the type of graph, which can be undirected unweighted, *UNONE*, or undirected weighted, *USYM*. With alternative *f*_*obj*_, we choose an objective function *f*_*obj*_ based on weighted density in the mining algorithm. to detect *best* dense subgraphs (the default function, *f*_*obj*_ = |*S* ∩ *C*|, is used with option *UNONE*). With *Sorting* we specify the sorting algorithm of adjacency lists; it can be by ID or by FREQUENCY. Finally, *Grouping* allows us to define how predicted complexes are built based on candidate complexes. Alternatives are *UNION*, which takes the union *C*_*x*_ ∪ *C*_*y*_ of the complexes where *OS*(*C*_*x*_, *C*_*y*_) > 0.8, and *NONE*, where predicted complexes are defined as the candidate complexes. Other parameters include the minimum size, *minSize*, of any complex, the type of dense subgraph (only clique or dense subgraphs) and an alternative mapping for input PPI networks.

**Table 4:** Parameter settings.

Options	Description
**Protein mapping (-m)**	
mappingFile	File mapping protein names to numeric ids
**Sorting (-r)**	
FREQUENCY	Sorting of adjacency list by frequency before building DAPG
ID	Sorting by id in adjacency list before building DAPG
**Grouping (-f)**: Predicted protein complex formation (*PC*) using *OS*(*C*_*x*_, *C*_*y*_) > 0.8	
UNION	*PC* = *C*_*x*_ ∪ *C*_*y*_
NONE	*C*_*x*_ and *C*_*y*_
**Graph Types (-g)**	
UNONE	Undirected-unweighted graph
USYM	Undirected-weighted graph
**Alternative** *f*_*obj*_ **(-w)**	
WEDGE	Select the dense subgraphs with higher weighted-edge-density
WDEGREE	Select the dense subgraphs with higher weighted-degree-density
FWEDGREE	Select the dense subgraphs with higher weighted-edge-density of *S* ∪ *C* induced subgraph.
FWEDGE	Select the dense subgraphs with higher weighted-degree-density of *S* ∪ *C* induced subgraph.

In order to compare our results we tried different node ordering (protein mapping) algorithms and different parameters in each experiment, given in the following format: DAPG*GTypeDM-rSorting-fGrouping* *(Protein Mapping)*. In this format *GType* can be *UU* (undirected unweighted) or *UW* (undirected weighted), *DM* can be any of the density measures; *Sorting* can be adjacency lists sorted by frequency (F) or ID (I); and *Grouping* is the way we group candidate complexes to generate predicted complexes, defined by the union set (U) or none (N).

Tables 5 and 6 show the performance of our algorithm with different node ordering algorithms (protein name to numeric id mapping) and total order function *ϕ* (ID, FREQUENCY). We observe that using *BFS* and *DFS* traversals provides best results in seven of the eight PPI networks we tested. Also the total order function *Sorting by ID* is very effective with these protein mappings, achieving best results in six of the eight PPI networks.

**Table 5:** Results of best clustering metrics (with CYC2008 gold standard) obtained with DAPG (with complexes of minimum size 3) using different node ordering algorithms and applying sorting (*ϕ* function) in small PPIs.

Network	Node ordering	Sorting	Complexes	FMeasure	Acc	MMR
Collins	First	FREQUENCY	620	0.7269	0.7226	0.7020
		ID	447	0.6782	0.7115	0.6749
	Lexicographic	FREQUENCY	623	0.7341	0.7259	0.7043
		ID	410	0.6983	0.7133	0.6469
	Random	FREQUENCY	626	0.7466	0.7225	0.7141
		ID	400	0.6517	0.7091	0.5986
	Degree	FREQUENCY	623	0.7280	0.7218	0.7036
		ID	484	0.6782	0.7160	0.6870
	BFS	FREQUENCY	633	0.7248	0.7234	**0.7183**
		ID	495	0.6578	0.7120	0.6739
	DFS	FREQUENCY	618	0.7289	0.7182	0.6999
		ID	509	0.6641	0.7106	0.6791
Krogan Core	First	FREQUENCY	651	0.6448	0.6178	0.4699
		ID	558	0.6191	0.6426	0.4814
	Lexicographic	FREQUENCY	627	0.6400	0.6391	0.4582
		ID	472	0.6027	0.6223	0.4321
	Random	FREQUENCY	627	0.6373	0.6199	0.4391
		ID	403	0.6030	0.5947	0.3863
	Degree	FREQUENCY	636	0.6516	0.6146	0.4688
		ID	564	0.6023	0.6060	0.4577
	BFS	FREQUENCY	614	0.6388	0.6279	0.4562
		ID	658	0.5784	0.6143	0.4991
	DFS	FREQUENCY	627	0.6353	0.6345	0.4556
		ID	649	0.6782	0.6242	**0.5059**
Krogan Extended	First	FREQUENCY	960	0.5142	0.6152	0.4226
		ID	864	0.4851	0.6248	0.4489
	Lexicographic	FREQUENCY	969	0.5294	0.6337	0.4321
		ID	732	0.4876	0.6120	0.4108
	Random	FREQUENCY	943	0.5250	0.6273	0.4328
		ID	809	0.4007	0.5816	0.3163
	Degree	FREQUENCY	947	0.5180	0.6172	0.4274
		ID	895	0.4720	0.6152	0.4212
	BFS	FREQUENCY	943	0.5303	0.6284	0.4217
		ID	970	0.4710	0.5947	0.4100
	DFS	FREQUENCY	967	0.5244	0.6232	0.4188
		ID	830	0.5411	0.6226	**0.4724**
Gavin	First	FREQUENCY	611	0.6516	0.7083	0.5809
		ID	641	0.5752	0.7055	0.5838
	Lexicographic	FREQUENCY	626	0.6491	0.7061	0.5827
		ID	503	0.6013	0.7028	0.5446
	Random	FREQUENCY	667	0.6441	0.7110	0.5908
		ID	474	0.5884	0.6901	0.5270
	Degree	FREQUENCY	612	0.6509	0.7089	0.5840
		ID	529	0.6097	0.6936	0.5592
	BFS	FREQUENCY	621	0.6454	0.7172	0.5819
		ID	715	0.6164	0.7135	**0.6079**
	DFS	FREQUENCY	620	0.6589	0.7148	0.5975
		ID	723	0.5500	0.6990	0.6006

**Table 6:** Results of best clustering metrics (with CYC2008 and CORUM references) obtained with DAPG (with complexes of minimum size 3) using different node ordering algorithms and applying sorting (*ϕ* function) in large PPIs.

Network	Node ordering	Sorting	Complexes	FMeasure	Acc	MMR
DIP-yeast	First	FREQUENCY	1,217	0.4000	0.5520	0.3615
		ID	1,141	0.3942	0.5416	0.3815
	Lexicographic	FREQUENCY	1,199	0.3872	0.5355	0.3550
		ID	1,085	0.4085	0.5565	0.3610
	Random	FREQUENCY	1,142	0.4070	0.5364	0.3491
		ID	909	0.3438	0.4808	0.2535
	Degree	FREQUENCY	1,212	0.3961	0.5489	0.3682
		ID	1,165	0.3835	0.5393	0.3560
	BFS	FREQUENCY	1,253	0.4197	0.5674	0.3751
		ID	1,242	0.3622	0.5551	0.3718
	DFS	FREQUENCY	1,210	0.4110	0.5450	0.3671
		ID	1,925	0.3830	0.5486	**0.4447**
Biogrid-yeast	First	FREQUENCY	5,025	0.1551	0.5691	0.3534
		ID	4,945	0.1444	0.5693	0.3371
	Lexicographic	FREQUENCY	4,999	0.1561	0.5727	0.3687
		ID	4,991	0.1740	0.5967	**0.3845**
	Random	FREQUENCY	5,017	0.1548	0.5718	0.3599
		ID	5,167	0.1108	0.5368	0.2614
	Degree	FREQUENCY	5,049	0.1533	0.5667	0.3439
		ID	5,004	0.1465	0.5677	0.3432
	BFS	FREQUENCY	4,977	0.1584	0.5741	0.3650
		ID	5,254	0.1047	0.5355	0.2711
	DFS	FREQUENCY	5,009	0.1570	0.5720	0.3627
		ID	4,950	0.1446	0.5800	0.3468
HPRD	First	FREQUENCY	2,437	0.3395	0.2140	0.1713
		ID	2,442	0.3200	0.2272	0.1743
	Lexicographic	FREQUENCY	2,430	0.3528	0.2103	0.1783
		ID	2,085	0.3542	0.2099	0.1643
	Random	FREQUENCY	2,430	0.3465	0.2121	0.1688
		ID	1,977	0.3464	0.1879	0.1326
	Degree	FREQUENCY	2,449	0.3401	0.2135	0.1706
		ID	2,412	0.3354	0.2127	0.1675
	BFS	FREQUENCY	2,441	0.3584	0.2139	0.1865
		ID	2,777	0.3685	0.2119	**0.2066**
	DFS	FREQUENCY	2,443	0.3484	0.2105	0.1668
		ID	2,313	0.3392	0.2340	0.1862
Biogrid-human	First	FREQUENCY	7,360	0.2380	0.2924	0.2387
		ID	7,200	0.2349	0.2825	0.2372
	Lexicographic	FREQUENCY	7,394	0.2474	0.2920	0.2405
		ID	7,313	0.2507	0.2738	0.2385
	Random	FREQUENCY	7,316	0.2492	0.2907	0.2332
		ID	7,663	0.2587	0.2732	0.2227
	Degree	FREQUENCY	7,375	0.2412	0.2920	0.2418
		ID	7,352	0.2352	0.2918	0.2374
	BFS	FREQUENCY	7,152	0.2453	0.2902	0.2354
		ID	8,144	0.2204	0.2854	0.2232
	DFS	FREQUENCY	7,409	0.2527	0.2917	**0.2539**
		ID	6,498	0.2309	0.2877	0.2228

We also explore the impact of adding random edges into a PPI networks. We present these results in Table 7. We observe that our scheme is robust based on the clustering metrics.

**Table 7:** Adding random interactions in yeast and human PPI networks (with CYC2008 and CORUM references) obtained with DAPG (with complexes of minimum size 3).

Network	Edges increased (%)	Complexes	FMeasure	Acc	MMR
Collins	5	522	0.7195	0.7102	0.6619
	10	501	0.7041	0.7270	0.6447
Krogan Core	5	611	0.6605	0.6165	0.4844
	10	591	0.6574	0.6290	0.4908
Krogan Extended	5	790	0.5287	0.6128	0.4430
	10	740	0.5506	0.6177	0.4410
Gavin	5	681	0.5996	0.7095	0.5879
	10	664	0.6072	0.7185	0.5733
DIP-yeast	5	1,989	0.3852	0.5471	0.4476
	10	2,011	0.3820	0.5499	0.4499
Biogrid-yeast	5	4,971	0.1686	0.5956	0.3787
	10	4,966	0.1615	0.5963	0.3737
HPRD	5	2,692	0.3582	0.2191	0.2000
	10	2,167	0.3462	0.2153	0.1897
Biogrid-human	5	7,047	0.2402	0.2998	0.2392
	10	6,857	0.2373	0.2925	0.2297

We show our best results in Table 8 using all gold standards. We obtain our best results using the objective function as *f*_*obj*_ = |*S* ∪ *C*| and only in DIP-yeast the degree density (WDEGREE) is better. We also obtain best results without merging or combining dense subgraphs, which is given by the grouping option NONE as described in Table 4.

**Table 8:** Our best results of clustering metrics obtained with DAPG (with complexes of minimum size 3).

Network	Algorithm	Complexes	Reference	FMeasure	Acc	MMR
Collins	DAPGU(BFS) rFfN	633				
			CYC2008	0.7248	0.7234	0.7183
			SGD	0.6037	0.5409	0.5956
			MIPS	0.5449	0.5417	0.4956
Krogan Core	DAPGU(DFS) rIfN	649				
			CYC2008	0.6782	0.6242	0.5059
			SGD	0.6266	0.4519	0.4153
			MIPS	0.4612	0.3793	0.3085
Krogan Extended	DAPGU(DFS) rIfN	830				
			CYC2008	0.5411	0.6226	0.4724
			SGD	0.4836	0.4400	0.3662
			MIPS	0.3724	0.3679	0.2747
Gavin	DAPGU(BFS) rIfN	715				
			CYC2008	0.6164	0.7135	0.6079
			SGD	0.5188	0.5270	0.4956
			MIPS	0.4376	0.4827	0.4304
DIP-yeast	DAPGUWD(DFS) rIfN	1,925				
			CYC2008	0.3830	0.5486	0.4447
			SGD	0.3473	0.4008	0.3620
			MIPS	0.2992	0.3475	0.3607
Biogrid-yeast	DAPGU(Lex) rIfN	4,991				
			CYC2008	0.1740	0.5967	0.3845
			SGD	0.1671	0.4627	0.3737
			MIPS	0.1292	0.3925	0.2994
HPRD	DAPGU(BFS) rIfN	2,777				
			CORUM	0.3685	0.2119	0.2066
			PCDq	0.3431	0.2992	0.1681
Biogrid-human	DAPGU(DFS) rFfN	7,409				
			CORUM	0.2527	0.2917	0.2539
			PCDq	0.1599	0.3495	0.1272

## Results

In this section we compare our best results with the state-of-the-art techniques such as ClusterONE [11], MCL [12], CFinder [10], GMFTP [26], MCODE [14], RNSC [15], SPICI [16], DCAFP [17], COREPEEL [18] and DSDCluster (winner of the challenge *Disease Module Identification DREAM Challenge* for subchallenge 1, https://www.synapse.org/#!Synapse:syn6156761/discussion/threadId=1073).

For each method we used the parameters that provided the best results. In the case of GMFTP we use default parameters (*τ* = 0.2, *K* = 1000, *λ* = 4, *T* = 400, *ρ* = 1*e* − 6) and set *repeat*_*times* = 10 instead of the default, which was 100. With this change we could actually get results in a little more than 12 hours for each PPI network. For CFinder the most sensible parameter is *t*, which is the allowed time to spend in the detection for clique search per node. We used *t* = 1 and *t* = 10 and took the best result. Since GMFTP took too much execution time for small PPI networks (over 12 hours) we did not try to run it with larger PPIs. Also, we were unable to execute CFinder with the two largest PPI networks, and with DCAFP we have a memory error with Biogrid-human, therefore we do not report results for these cases. The main parameter for executing DSDCluster is the number of clusters (*K*). We executed DSDCluster with *K* between 100 and 700, increasing by 100 in Collins, Krogan Core, Krogan Extended, and Gavin. In DIP-yeast we reach *K* = 1600. For Bigrid-yeast, HPRD and Biogrid-human we define *K* = 500, 1000, 1500, 2000, 2500. We obtain the best results with *K* = 200 in Collins, *K* = 500 in Krogan Core, *K* = 700 in Krogan Extended, *K* = 500 in Gavin, *K* = 1200 in DIP-yeast, *K* = 1000 in Biogrid-yeast, *K* = 2000 in HPRD, and *K* = 2500 in Biogrid-human.

Tables 9 to 14 show our results compared with the state-of-the-art techniques available for protein complex prediction for yeast. Similarly, Table 15 show the results for human. We evaluated clustering metrics and biological metrics. We observed that we are able to obtain the best MMR measure in Collins, Gavin, DIP-yeast and Biogrid-yeast PPI networks using the three gold standards and our combinations. In the Krogan Core PPI we obtain the second best after GMFTP, which is the best for the three gold standards, but we are better in the combined references. In the Krogan Extended PPI we are best using CYC2008, GMFTP is best with SGD and COREPEEL is best in MIPS, in the merged gold standards COREPEEL is the best, and we are second. We also observed that, for most human PPIs, COREPEEL is the best and we are second. We also report execution times, where all methods were executed locally, except COREPEEL, which provide the execution through its web site and report execution time as a result. SPICI is the fastest method.

**Table 9:** Performance comparison results of clustering and biological metrics in Collins.

Approach	#C	FM	Acc	MMR	GoSim	Coloc.	SC	Time(s)
**Collins**		**CYC2008**						
DAPG	633	0.7248	0.7234	**0.7183**	0.9692	0.7692	0.9435	2.36
GMFTP	189	0.7631	0.7858	0.6410	0.9542	0.7489	0.9052	> 12hrs.
ClusterONE	187	0.6940	0.7677	0.5711	0.9211	0.7124	0.8225	1.37
MCL	195	0.6897	0.7635	0.5729	0.9268	0.7310	0.8823	0.74
CFinder	113	0.6583	0.6518	0.4361	0.8641	0.6173	0.9027	119.54
DCAFP	880	**0.8433**	0.6784	0.5575	0.9386	0.7212	0.9234	231.18
RNSC	178	0.6980	0.7756	0.5812	0.9313	0.7397	0.8930	1.42
MCODE	93	0.6233	0.6035	0.3213	0.8750	0.6345	0.9125	0.52
SPICI	104	0.6579	0.7145	0.4115	0.9476	0.7546	0.9214	0.14
COREPEEL	458	0.6751	0.7037	0.6718	0.9501	0.7377	0.9334	0.23
DSDCluster	142	0.4626	0.6065	0.2863	0.9179	0.7533	0.8943	41.93
		**SGD**						
DAPG	633	0.6037	0.5409	**0.5956**				
GMFTP	189	0.6795	0.5988	0.5295				
ClusterONE	187	0.5817	0.6017	0.4357				
MCL	195	0.6039	0.5885	0.4500				
CFinder	113	0.5126	0.5143	0.3215				
DCAFP	880	**0.7091**	0.5103	0.4959				
RNSC	178	0.6207	0.5899	0.4432				
MCODE	93	0.5048	0.5050	0.2430				
SPICI	104	0.5845	0.5456	0.3096				
COREPEEL	458	0.5646	0.5251	0.5151				
DSDCluster	142	0.3838	0.4595	0.2124				
		**MIPS**						
DAPG	633	0.5449	0.5417	**0.4956**				
GMFTP	189	0.5356	0.5338	0.4269				
ClusterONE	187	0.5517	0.5439	0.4110				
MCL	195	0.4742	0.5070	0.3856				
CFinder	113	0.5023	0.4430	0.3042				
DCAFP	880	**0.6930**	0.5275	0.4302				
RNSC	178	0.5147	0.5182	0.4070				
MCODE	93	0.5532	0.4804	0.2808				
SPICI	104	0.5500	0.5046	0.3063				
COREPEEL	458	0.4739	0.5271	0.4402				
DSDCluster	142	0.3838	0.4595	0.2124				
		**CYC2008**, **SGD**						
DAPG	633	0.7157	0.5591	**0.5837**				
GMFTP	189	0.7202	0.5846	0.4549				
ClusterONE	187	0.6325	0.5842	0.3955				
MCL	195	0.6424	0.5709	0.4034				
CFinder	113	0.5348	0.5005	0.2914				
DCAFP	880	**0.8193**	0.5332	0.5008				
RNSC	178	0.6624	0.5794	0.4044				
MCODE	93	0.5508	0.4745	0.2274				
SPICI	104	0.5772	0.5343	0.2743				
COREPEEL	458	0.6667	0.5375	0.5032				
DSDCluster	142	0.2834	0.4295	0.1688				
		**CYC2008**, **SGD**, **MIPS**						
DAPG	633	0.7101	0.5480	**0.5723**				
GMFTP	189	0.7143	0.5770	0.4376				
ClusterONE	187	0.6265	0.5765	0.3825				
MCL	195	0.6424	0.5616	0.3903				
CFinder	113	0.5201	0.4907	0.2803				
DCAFP	880	**0.8119**	0.5253	0.4891				
RNSC	178	0.6581	0.5713	0.3939				
MCODE	93	0.5424	0.4700	0.2185				
SPICI	104	0.5645	0.5279	0.2640				
COREPEEL	458	0.6620	0.5269	0.4961				
DSDCluster	142	0.4407	0.4628	0.2101				

**Table 10:** Performance comparison results of clustering and biological metrics in Krogan Core.

Approach	#C	FM	Acc	MMR	GoSim	Coloc.	SC	Time(s)
**Krogan Core**		**CYC2008**						
DAPG	649	0.6782	0.6242	0.5059	0.8976	0.7099	0.8533	2.19
GMFTP	287	0.6079	0.7731	**0.5370**	0.8524	0.6741	0.7026	> 12hrs.
ClusterONE	411	0.5844	0.7409	0.5065	0.7937	0.6542	0.6830	1.65
MCL	377	0.4226	0.7362	0.4119	0.6794	0.5975	0.6072	8.62
CFinder	113	0.4719	0.5477	0.2783	0.7203	0.5329	0.7653	0.33
DCAFP	384	**0.8494**	0.5814	0.3278	0.8587	0.7269	0.9043	640.06
RNSC	293	0.4732	0.6951	0.4378	0.7970	0.6818	0.6110	0.68
MCODE	83	0.4615	0.5282	0.1829	0.7807	0.6345	0.7271	5.68
SPICI	133	0.5714	0.6581	0.3293	0.9076	0.7132	0.8125	0.18
COREPEEL	723	0.6042	0.6032	0.4869	0.8733	0.7086	0.7869	0.24
DSDCluster	368	0.4208	0.7044	0.4064	0.6579	0.5667	0.5667	121.96
		**SGD**						
DAPG	649	0.6266	0.4519	0.4153				
GMFTP	287	0.5536	0.5550	**0.4270**				
ClusterONE	411	0.5261	0.5520	0.3833				
MCL	377	0.3680	0.5336	0.2970				
CFinder	113	0.4014	0.3994	0.2051				
DCAFP	384	**0.7637**	0.4234	0.2842				
RNSC	293	0.4340	0.5056	0.3220				
MCODE	83	0.3745	0.3950	0.1324				
SPICI	133	0.5300	0.4881	0.2604				
COREPEEL	723	0.5497	0.4406	0.3967				
DSDCluster	368	0.3804	0.5041	0.3137				
		**MIPS**						
DAPG	649	0.4612	0.3793	0.3085				
GMFTP	287	0.3990	0.4597	**0.3479**				
ClusterONE	411	0.3443	0.4363	0.3356				
MCL	377	0.2729	0.4362	0.2681				
CFinder	113	0.3030	0.3417	0.1638				
DCAFP	384	**0.6396**	0.3835	0.2731				
RNSC	293	0.2843	0.4142	0.2560				
MCODE	83	0.3415	0.3625	0.1257				
SPICI	133	0.3443	0.4000	0.1952				
COREPEEL	723	0.4118	0.3699	0.2829				
DSDCluster	368	0.2672	0.4123	0.2720				
		**CYC2008**, **SGD**						
DAPG	649	0.6760	0.4206	**0.4115**				
GMFTP	287	0.5921	0.5327	0.3682				
ClusterONE	411	0.5868	0.5284	0.3526				
MCL	377	0.4007	0.5140	0.2677				
CFinder	113	0.3939	0.3810	0.1849				
DCAFP	384	**0.7929**	0.4048	0.2797				
RNSC	293	0.4555	0.4863	0.2878				
MCODE	83	0.3436	0.3774	0.1149				
SPICI	133	0.5128	0.4592	0.2164				
COREPEEL	723	0.6053	0.4073	0.3943				
DSDCluster	368	0.4135	0.4899	0.2805				
		**CYC2008**, **SGD**, **MIPS**						
DAPG	649	0.6734	0.4116	**0.4022**				
GMFTP	287	0.5914	0.5251	0.3578				
ClusterONE	411	0.5918	0.5196	0.3487				
MCL	377	0.4007	0.5041	0.2617				
CFinder	113	0.3871	0.3737	0.1788				
DCAFP	384	**0.7756**	0.3951	0.2752				
RNSC	293	0.4590	0.4772	0.2836				
MCODE	83	0.3467	0.3678	0.1122				
SPICI	133	0.5000	0.4513	0.2094				
COREPEEL	723	0.6046	0.3981	0.3883				
DSDCluster	368	0.4885	0.4799	0.2692				

**Table 11:** Performance comparison results of clustering and biological metrics in Krogan Extended.

Approach	#C	FM	Acc	MMR	GoSim	Coloc.	SC	Time(s)
**Krogan Extended**		**CYC2008**						
DAPG	830	0.5411	0.6226	**0.4724**	0.8268	0.6798	0.6783	8.33
GMFTP	364	0.4510	0.7389	0.4509	0.7634	0.6165	0.5792	> 12 hrs.
ClusterONE	402	0.5751	0.7043	0.4551	0.7960	0.6546	0.6741	2.18
MCL	480	0.3328	0.7154	0.3113	0.5977	0.5231	0.4987	19.50
CFinder	118	0.2993	0.4126	0.1682	0.6154	0.4466	0.6365	1.43
DCAFP	519	**0.7302**	0.5928	0.3356	0.8924	0.7442	0.7343	750.23
RNSC	326	0.3589	0.6657	0.3322	0.7233	0.6399	0.4923	0.24
MCODE	55	0.2807	0.4365	0.1044	0.6687	0.5143	0.7872	13.12
SPICI	147	0.5364	0.6370	0.3126	0.8700	0.6971	0.7172	0.10
COREPEEL	1223	0.4842	0.6236	0.4564	0.8302	0.6886	0.6884	0.26
DSDCluster	530	0.3105	0.6619	0.3250	0.5856	0.5212	0.4301	480.08
		**SGD**						
DAPG	830	0.4836	0.4400	0.3662				
GMFTP	364	0.4400	0.5221	0.3532				
ClusterONE	402	0.4992	0.5187	0.3259				
MCL	480	0.2708	0.5040	0.2121				
CFinder	118	0.2531	0.3155	0.1312				
DCAFP	519	**0.6551**	0.4244	0.2714				
RNSC	326	0.3230	0.4754	0.2455				
MCODE	55	0.2162	0.3157	0.0761				
SPICI	147	0.4969	0.4655	0.2424				
COREPEEL	1,223	0.4350	0.4486	**0.3762**				
DSDCluster	530	0.2639	0.4715	0.2408				
		**MIPS**						
DAPG	830	0.3724	0.3679	0.2747				
GMFTP	364	0.3056	0.4430	**0.2980**				
ClusterONE	402	0.3417	0.4184	0.2904				
MCL	480	0.2065	0.4075	0.1928				
CFinder	118	0.2022	0.2491	0.1059				
DCAFP	519	**0.5392**	0.3795	0.2451				
RNSC	326	0.2495	0.3927	0.2165				
MCODE	55	0.2079	0.2938	0.0608				
SPICI	147	0.3286	0.3804	0.1847				
COREPEEL	1,223	0.3325	0.3787	0.2806				
DSDCluster	530	0.1898	0.3749	0.2061				
		**CYC2008**, **SGD**						
DAPG	830	0.5344	0.4076	0.3603				
GMFTP	364	0.4582	0.5000	0.2974				
ClusterONE	402	0.5606	0.4954	0.3013				
MCL	480	0.3145	0.4906	0.1970				
CFinder	118	0.2398	0.2964	0.1127				
DCAFP	519	**0.7045**	0.4074	0.2699				
RNSC	326	0.3517	0.4610	0.2186				
MCODE	55	0.2105	0.3036	0.0653				
SPICI	147	0.4833	0.4416	0.2054				
COREPEEL	1,223	0.4937	0.4151	**0.3661**				
DSDCluster	530	0.3009	0.4538	0.2177				
		**CYC2008**, **SGD**, **MIPS**						
DAPG	830	0.5362	0.3996	0.3563				
GMFTP	364	0.4577	0.4897	0.2905				
ClusterONE	402	0.5714	0.4859	0.2985				
MCL	480	0.3169	0.4788	0.1930				
CFinder	118	0.2376	0.2894	0.1096				
DCAFP	519	**0.7022**	0.3968	0.2720				
RNSC	326	0.3531	0.4508	0.2122				
MCODE	55	0.2018	0.2965	0.0628				
SPICI	147	0.4796	0.4316	0.1989				
COREPEEL	1,223	0.4943	0.4041	**0.3612**				
DSDCluster	530	0.3018	0.4939	0.2093				

**Table 12:** Performance comparison results of clustering and biological metrics in Gavin.

Approach	#C	FM	Acc	MMR	GoSim	Coloc.	SC	Time(s)
**Gavin**		**CYC2008**						
DAPG	715	0.6164	0.7135	**0.6079**	0.8750	0.6687	0.8041	1.66
GMFTP	242	0.6096	0.7705	0.5861	0.8586	0.6761	0.7561	> 12hrs
ClusterONE	194	0.6854	0.7498	0.5378	0.8934	0.6810	0.8367	1.41
MCL	254	0.5372	0.7435	0.4828	0.7865	0.6342	0.7124	2.01
CFinder	183	0.4466	0.6210	0.3391	0.7335	0.5370	0.6412	598.84
DCAFP	804	**0.7118**	0.6296	0.4416	0.8855	0.6626	0.7843	133.79
RNSC	241	0.5556	0.7551	0.5106	0.8188	0.6566	0.7135	0.056
MCODE	107	0.5281	0.6092	0.2547	0.8081	0.5954	0.7982	11.28
SPICI	91	0.6574	0.5905	0.3381	0.8965	0.7458	0.8972	0.09
COREPEEL	690	0.5795	0.6998	0.5686	0.8643	0.6883	0.7753	0.15
DSDCluster	265	0.5390	0.6918	0.4662	0.8101	0.6587	0.6603	63.70
		**SGD**						
DAPG	715	0.5188	0.5270	**0.4956**				
GMFTP	242	0.5393	0.5842	0.4448				
ClusterONE	194	0.5855	0.5702	0.3974				
MCL	254	0.4641	0.5502	0.3510				
CFinder	183	0.3529	0.4794	0.2526				
DCAFP	804	**0.6393**	0.4849	0.4062				
RNSC	241	0.4638	0.5703	0.3731				
MCODE	107	0.3964	0.4763	0.1784				
SPICI	91	0.5481	0.4509	0.2473				
COREPEEL	690	0.4692	0.5067	0.4643				
DSDCluster	265	0.4543	0.5102	0.3419				
		**MIPS**						
DAPG	715	0.4376	0.4827	**0.4304**				
GMFTP	242	0.4602	0.5240	0.4206				
ClusterONE	194	0.4846	0.4981	0.3728				
MCL	254	0.3746	0.4983	0.3266				
CFinder	183	0.3559	0.4382	0.2618				
DCAFP	804	**0.5552**	0.4628	0.3732				
RNSC	241	0.4012	0.4990	0.3560				
MCODE	107	0.4038	0.4362	0.2007				
SPICI	91	0.4375	0.3737	0.2182				
COREPEEL	690	0.4049	0.4679	0.4262				
DSDCluster	265	0.3552	0.4520	0.3092				
		**CYC2008**, **SGD**						
DAPG	715	0.6163	0.5137	**0.4893**				
GMFTP	242	0.6114	0.5686	0.4197				
ClusterONE	194	0.6566	0.5476	0.3706				
MCL	254	0.5168	0.5440	0.3296				
CFinder	183	0.3988	0.4768	0.2365				
DCAFP	804	**0.7074**	0.4606	0.4016				
RNSC	241	0.5308	0.5542	0.3452				
MCODE	107	0.4471	0.4601	0.1707				
SPICI	91	0.5992	0.4303	0.2357				
COREPEEL	690	0.5752	0.5000	0.4607				
DSDCluster	265	0.5255	0.4995	0.3246				
		**CYC2008**, **SGD**, **MIPS**						
DAPG	715	0.6177	0.5022	**0.4840**				
GMFTP	242	0.6078	0.5570	0.4070				
ClusterONE	194	0.6465	0.5358	0.3549				
MCL	254	0.5103	0.5308	0.3174				
CFinder	183	0.3851	0.4637	0.2234				
DCAFP	804	**0.7024**	0.4508	0.4001				
RNSC	241	0.5255	0.5430	0.3314				
MCODE	107	0.4479	0.4488	0.1651				
SPICI	91	0.5868	0.4195	0.2273				
COREPEEL	690	0.5749	0.4882	0.4503				
DSDCluster	265	0.5243	0.4891	0.3121				

**Table 13:** Performance comparison results of clustering and biological metrics in DIP-yeast.

Approach	#C	FM	Acc	MMR	GoSim	Coloc.	SC	Time(s)
**DIP-yeast**		**CYC2008**						
DAPG	1,925	0.3830	0.5486	**0.4447**	0.8133	0.6664	0.8082	6.23
ClusterONE	1,042	0.2436	0.6236	0.2794	0.6353	0.5682	0.4432	1.44
MCL	598	0.2685	0.6259	0.2389	0.5986	0.5355	0.4523	2.31
CFinder	198	0.2721	0.4272	0.1598	0.5843	0.4173	0.4371	3.02
DCAFP	492	**0.7212**	0.5631	0.2972	0.8897	0.7187	0.8289	3,848.32
RNSC	517	0.0108	0.2966	0.0063	0.8001	0.6218	0.1043	0.53
MCODE	78	0.2007	0.3734	0.0663	0.6784	0.4546	0.8023	33.42
SPICI	517	0.3007	0.5826	0.2394	0.6650	0.5697	0.6342	0.12
COREPEEL	742	0.5160	0.5679	0.3239	0.8287	0.6500	0.8277	0.16
DSDCluster	645	0.2787	0.5688	0.2606	0.6233	0.5442	0.4728	2,520.67
		**SGD**						
DAPG	1,925	0.3473	0.4008	**0.3620**				
ClusterONE	1,042	0.2236	0.4684	0.2179				
MCL	598	0.2377	0.4454	0.1818				
CFinder	198	0.2133	0.3171	0.1145				
DCAFP	492	**0.6089**	0.4043	0.2329				
RNSC	517	0.0102	0.2116	0.0053				
MCODE	78	0.1641	0.2784	0.0530				
SPICI	517	0.2884	0.4322	0.1859				
COREPEEL	742	0.4854	0.4153	0.2761				
DSDCluster	645	0.2503	0.4079	0.2109				
		**MIPS**						
DAPG	1,925	0.2992	0.3475	**0.3607**				
ClusterONE	1,042	0.1422	0.3697	0.1865				
MCL	598	0.1695	0.3598	0.1713				
CFinder	198	0.1739	0.2584	0.1069				
DCAFP	492	**0.6181**	0.3727	0.2649				
RNSC	517	0.0029	0.1717	0.0014				
MCODE	78	0.1562	0.2572	0.0451				
SPICI	517	0.2101	0.3561	0.1759				
COREPEEL	742	0.3938	0.3619	0.2428				
DSDCluster	645	0.1776	0.3525	0.1768				
		**CYC2008**, **SGD**						
DAPG	1,925	0.4138	0.3769	**0.3654**				
ClusterONE	1,042	0.2690	0.4441	0.2076				
MCL	598	0.2835	0.4358	0.1725				
CFinder	198	0.2366	0.3053	0.1045				
DCAFP	492	**0.6743**	0.3806	0.2282				
RNSC	517	0.0092	0.1991	0.0040				
MCODE	78	0.1691	0.2663	0.0485				
SPICI	517	0.3041	0.4126	0.1651				
COREPEEL	742	0.5395	0.3871	0.2695				
DSDCluster	645	0.2866	0.3966	0.1896				
		**CYC2008**, **SGD**, **MIPS**						
DAPG	1,925	0.4213	0.3684	**0.3684**				
ClusterONE	1,042	0.2718	0.4368	0.2009				
MCL	598	0.2832	0.4269	0.1646				
CFinder	198	0.2389	0.3003	0.1039				
DCAFP	492	**0.6704**	0.3723	0.2321				
RNSC	517	0.0089	0.1938	0.0037				
MCODE	78	0.1606	0.2588	0.0462				
SPICI	517	0.3131	0.4042	0.1620				
COREPEEL	742	0.5437	0.3788	0.2711				
DSDCluster	645	0.2914	0.3894	0.1840				

**Table 14:** Performance comparison results of clustering and biological metrics in Biogrid-yeast.

Approach	#C	FM	Acc	MMR	GoSim	Coloc.	SC	Time(s)
**Biogrid-yeast**		**CYC2008**						
DAPG	4,991	0.1740	0.5967	**0.3845**	0.7143	0.5410	0.6524	144.58
ClusterONE	369	0.3132	0.5426	0.1599	0.8241	0.6370	0.4203	42.74
MCL	136	0.0919	0.2872	0.0303	0.5624	0.5794	0.5156	63.23
DCAFP	1,545	**0.4250**	0.4642	0.2846	0.6590	0.4149	0.9043	20,063.2
RNSC	755	0.1264	0.5868	0.1301	0.6680	0.5822	0.4351	128.29
MCODE	24	0.0077	0.1220	0.0014	0.4582	0.3355	0.7523	5,562.32
SPICI	389	0.1618	0.5154	0.0839	0.6317	0.4797	0.5434	0.82
COREPEEL	5,406	0.2048	0.5490	0.3412	0.7356	0.5611	0.6918	23.02
DSDCluster	557	0.3019	0.5576	0.2282	0.6414	0.5340	0.6879	4.5 hrs.
		**SGD**						
DAPG	4,977	0.1484	0.4386	**0.3405**				
ClusterONE	369	0.3062	0.4341	0.1438				
MCL	136	0.0852	0.2313	0.0296				
DCAFP	1,545	**0.4048**	0.3729	0.2731				
RNSC	755	0.1263	0.4685	0.1174				
MCODE	24	0.0067	0.0885	0.0012				
SPICI	389	0.1469	0.4156	0.0680				
COREPEEL	5,406	0.1654	0.4116	0.3038				
DSDCluster	557	0.2686	0.4144	0.1885				
		**MIPS**						
DAPG	4,977	0.1038	0.3787	**0.2700**				
ClusterONE	369	0.2094	0.3769	0.1096				
MCL	136	0.0559	0.1943	0.0221				
DCAFP	1,545	**0.3666**	0.3819	0.2667				
RNSC	755	0.0905	0.4016	0.1026				
MCODE	24	0.0094	0.1074	0.0017				
SPICI	389	0.1117	0.3861	0.0684				
COREPEEL	5,406	0.1437	0.3570	0.2431				
DSDCluster	557	0.1951	0.3510	0.1597				
		**CYC2008**, **SGD**						
DAPG	4,977	0.1834	0.4098	**0.3294**				
ClusterONE	369	0.3412	0.4167	0.1332				
MCL	136	0.0797	0.2113	0.0247				
DCAFP	1,545	**0.4578**	0.3507	0.2552				
RNSC	755	0.1469	0.4610	0.1057				
MCODE	24	0.0050	0.0875	0.0008				
SPICI	389	0.1603	0.3964	0.0614				
COREPEEL	5,406	0.2164	0.3802	0.2935				
DSDCluster	557	0.3177	0.4083	0.1783				
		**CYC2008**, **SGD**, **MIPS**						
DAPG	4,977	0.1885	0.4032	**0.3219**				
ClusterONE	369	0.3342	0.4065	0.1281				
MCL	136	0.0795	0.2055	0.0236				
DCAFP	1,545	**0.4569**	0.3430	0.2593				
RNSC	755	0.1447	0.4518	0.0999				
MCODE	24	0.0047	0.0857	0.0008				
SPICI	389	0.1585	0.3876	0.0590				
COREPEEL	5,406	0.2217	0.3751	0.2897				
DSDCluster	557	0.3131	0.4003	0.1691				

**Table 15:** Performance comparison results of clustering and biological metrics in HPRD and Biogrid-human.

Approach	#C	FM	Acc	MMR	GoSim	Coloc.	SC	Time(s)
**HPRD**		**PCDq**						
DAPG	2,777	0.3431	0.2992	0.1681	0.9225	0.4192	0.6564	30.78
ClusterONE	2,186	0.2923	0.5122	0.1718	0.7735	0.4106	0.3114	4.6
MCL	1,248	0.2167	0.4717	0.1120	0.7430	0.3831	0.4150	10.39
CFinder	416	0.1637	0.2935	0.0598	0.6283	0.3284	0.2383	12.42
DCAFP	123	0.1185	0.1654	0.0086	0.8532	0.3440	0.8848	25,470.12
RNSC	1,081	0.2250	0.4445	0.1122	0.8235	0.4241	0.3862	2.32
MCODE	16	0.0170	0.1003	0.0041	0.8033	0.5806	0.6553	10.23
SPICI	722	0.2410	0.4148	0.0835	0.7856	0.3801	0.4510	0.82
COREPEEL	3,420	**0.3577**	0.2943	**0.1852**	0.9249	0.4074	0.6667	1.01
DSDCluster	1,247	0.2012	0.4181	0.0994	0.7389	0.3874	0.5405	3.8 hrs.
		**CORUM**						
DAPG	2,777	0.3685	0.2119	0.2066				
ClusterONE	2,186	0.1348	0.3162	0.0730				
MCL	1,248	0.1048	0.3042	0.0488				
CFinder	416	0.0769	0.1982	0.0270				
DCAFP	123	0.1490	0.1460	0.0270				
RNSC	1,081	0.1234	0.2773	0.0565				
MCODE	16	0.0154	0.0786	0.0047				
SPICI	722	0.1095	0.2566	0.0357				
COREPEEL	3,420	**0.4017**	0.2131	**0.2360**				
DSDCluster	1,247	0.1056	0.2671	0.0510				
		**CORUM**, **PCDq**						
DAPG	2,777	0.4757	0.1987	0.1788				
ClusterONE	2,186	0.2887	0.3485	0.1101				
MCL	1,248	0.1936	0.3233	0.0701				
CFinder	416	0.1166	0.2036	0.0368				
DCAFP	123	0.0898	0.1161	0.0155				
RNSC	1,081	0.2080	0.3010	0.0743				
MCODE	16	0.0094	0.0652	0.0027				
SPICI	722	0.1946	0.2761	0.0506				
COREPEEL	3,420	**0.5168**	0.1970	**0.2033**				
DSDCluster	1,247	0.1884	0.2837	0.0661				
**Biogrid Human**		**PCDq**						
DAPG	7,409	0.1599	0.3495	0.1272	0.8213	0.4041	0.5443	620.32
ClusterONE	4,254	0.0863	0.4802	0.0653	0.6476	0.4008	0.2532	201.32
MCL	1,433	0.0431	0.3594	0.0190	0.6225	0.3695	0.2392	54.21
RNSC	2,194	0.0774	0.4491	0.0502	0.8235	0.3971	0.2206	35.23
MCODE	20	0.0063	0.0883	0.0013	0.8312	0.3695	0.5262	475.23
SPICI	1,063	0.0803	0.3784	0.0263	0.6763	0.3729	0.3829	1.01
COREPEEL	9,772	**0.1995**	0.3200	**0.1550**	0.8468	0.4059	0.5782	10.83
DSDCluster	1,593	0.0610	0.3673	0.0307	0.6344	0.3601	0.4148	5.5 hrs.
		**CORUM**						
DAPG	7,409	0.2527	0.2917	0.2539				
ClusterONE	4,254	0.0529	0.3625	0.0417				
MCL	1,433	0.0403	0.2610	0.0179				
RNSC	2,194	0.0637	0.3632	0.0418				
MCODE	20	0.0105	0.1046	0.0032				
SPICI	1,063	0.0643	0.3013	0.0235				
COREPEEL	9,772	**0.3477**	0.2778	**0.3063**				
DSDCluster	1,593	0.0824	0.3118	0.0409				
		**CORUM**, **PCDq**						
DAPG	7,409	0.3002	0.2585	0.1847				
ClusterONE	4,254	0.1020	0.3709	0.0485				
MCL	1433	0.0512	0.2655	0.0165				
RNSC	2,194	0.0921	0.3596	0.0402				
MCODE	20	0.0069	0.0878	0.0018				
SPICI	1,063	0.0836	0.2899	0.0217				
COREPEEL	9,772	**0.3965**	0.2414	**0.2250**				
DSDCluster	1,593	0.0848	0.2904	0.0305				

### Evaluating overlap on predicted complexes

**Fig 2:**
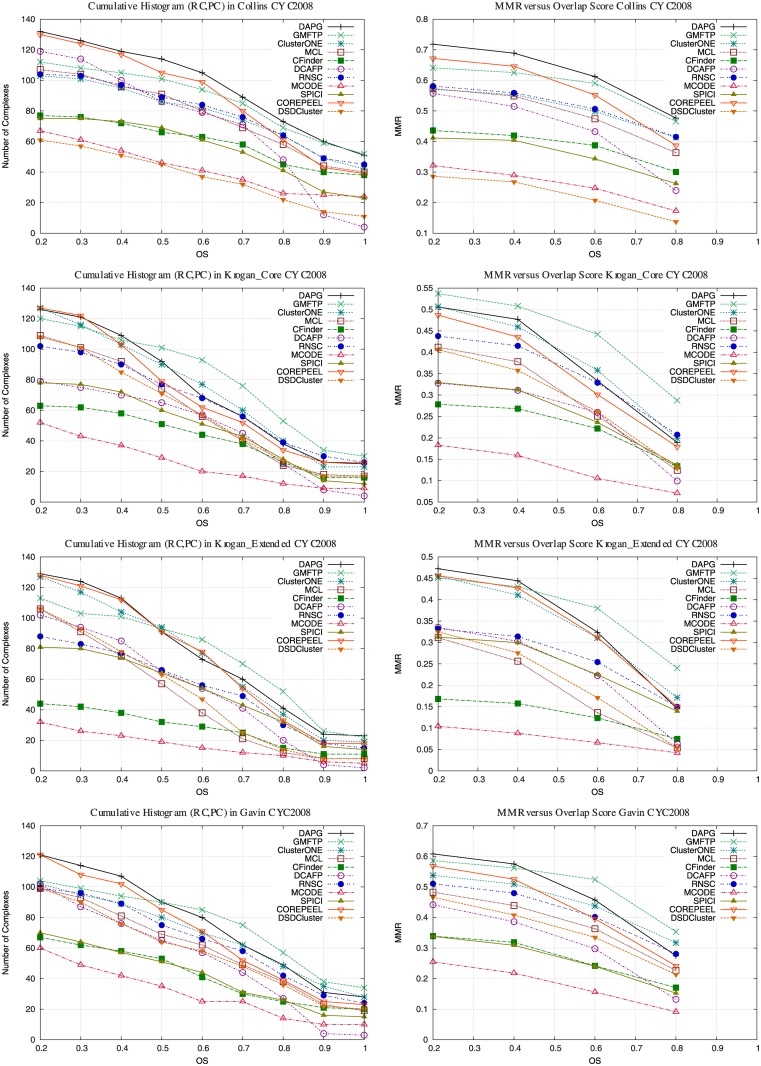
Cumulative histogram for predicted complexes matches with reference complexes based on MMR on small PPIs. Matching predicted complexes to reference complexes cumulative histogram for various yeast PPI networks and references CYC2008. Figures on right column show how MMR varies when changing the overlap score.

**Fig 3:**
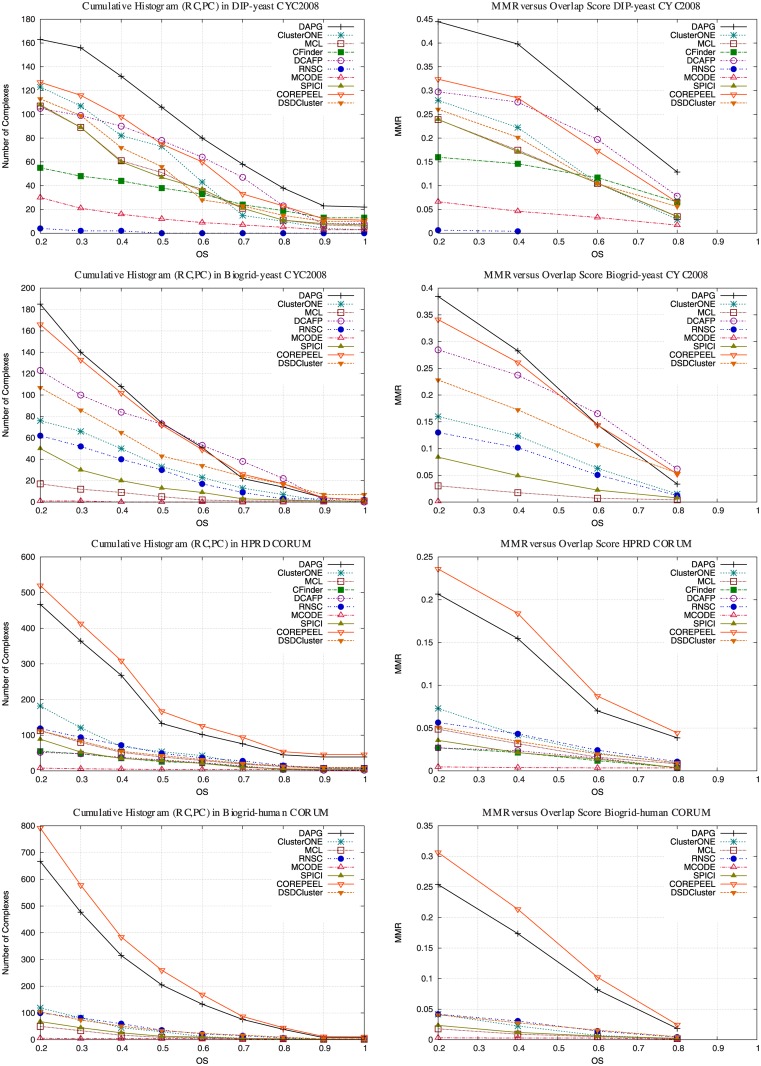
Cumulative histogram for predicted complexes matches with reference complexes based on MMR on large PPIs. Matching predicted complexes to reference complexes cumulative histogram for a large yeast PPI network using references CYC2008, and two Human PPI networks using gold standard CORUM. Figures on right column show how MMR varies when changing the overlap score.

In this section we evaluate how well protein complexes in gold standards are matched with predicted complexes. We first evaluated and compared the protein complex overlap as described earlier using cumulative histograms. We compute the cumulative histogram of all pairs of reference complex and predicted complex (*c*_*i*_, *pc*_*j*_) obtained when computing the MMR (where *OS*(*c*_*i*_, *pc*_*j*_) ≥ 0.2). We also compute the MMR varying the overlap score threshold. Figs 2 and 3 (left column) shows the cumulative histogram for overlap between predicted and reference complexes for all PPIs. Figs 2 and 3 (right column) shows the MMR for different overlap scores. We observed that DAPG is best in Collins and DIP-yeast, although, we did not tried GMFTP in DIP-yeast because it was several orders of magnitude slower than DAPG in smaller PPIs (as seen in Tables 9 to 12). We also show that DAPG has the best MMR results considering different overlap scores.

In addition, we show in Table 16 the number of predicted complexes that are correctly predicted (*OS* = 1.0) by DAPG and the state-of-the-art methods. We observed that GMFTP provides the greatest number of perfect matches in all small yeast references, except in Krogan Extended, where we get one more complex. We are second best, except on Krogan Core (where RNSC gets one more complex) and in Biogrid-yeast (where DSDCluster identifies 5 more complexes than DAPG, COREPEEL and RNSC). Also, in the human PPIs, we are second after COREPEEL.

**Table 16:** Number of predicted complexes with perfect matching with complexes in references (CYC2008 and CORUM) (*OS* = 1.0).

**Small networks**				
**Approach**	**Collins**	**Krogan Core**	**Krogan Extended**	**Gavin**
DAPG	51	25	23	28
GMFTP	52	30	22	34
ClusterONE	42	23	19	28
MCL	40	17	8	19
CFinder	38	16	11	20
DCAFP	4	4	3	3
RNSC	45	26	15	24
MCODE	24	9	5	10
SPICI	23	12	18	23
COREPEEL	39	26	18	23
DSDCluster	11	17	8	20
**larger networks**				
**Approach**	**DIP-yeast**	**Biogrid-yeast**	**HPRD**	**Biogrid-human**
DAPG	22	2	39	8
ClusterONE	3	1	8	1
MCL	6	1	7	2
CFinder	13	-	4	-
DCAFP	8	0	2	-
RNSC	0	2	8	1
MCODE	3	0	2	1
SPICI	7	1	1	1
COREPEEL	11	2	46	11
DSDCluster	10	7	10	3

We also compared our algorithm with the most competitive methods, GMFTP and COREPEEL, based on some patterns we detected in the PPIs. We considered the four following complexes for yeast, described in the gold standard CYC2008.

- HIR complex: HIR1, HIR2, HIR3, HPC2
- Phosphatidylinositol (PtdIns) 3-kinase complex (functions in CPY sorting): VPS15, VPS30, VPS34, VPS38
- AP-3 Adaptor complex: APL5, APL6, APM3, APS3
- EKC/KEOPS complex: CGI121, BUD32, GON7, KAE1

**Fig 4:**
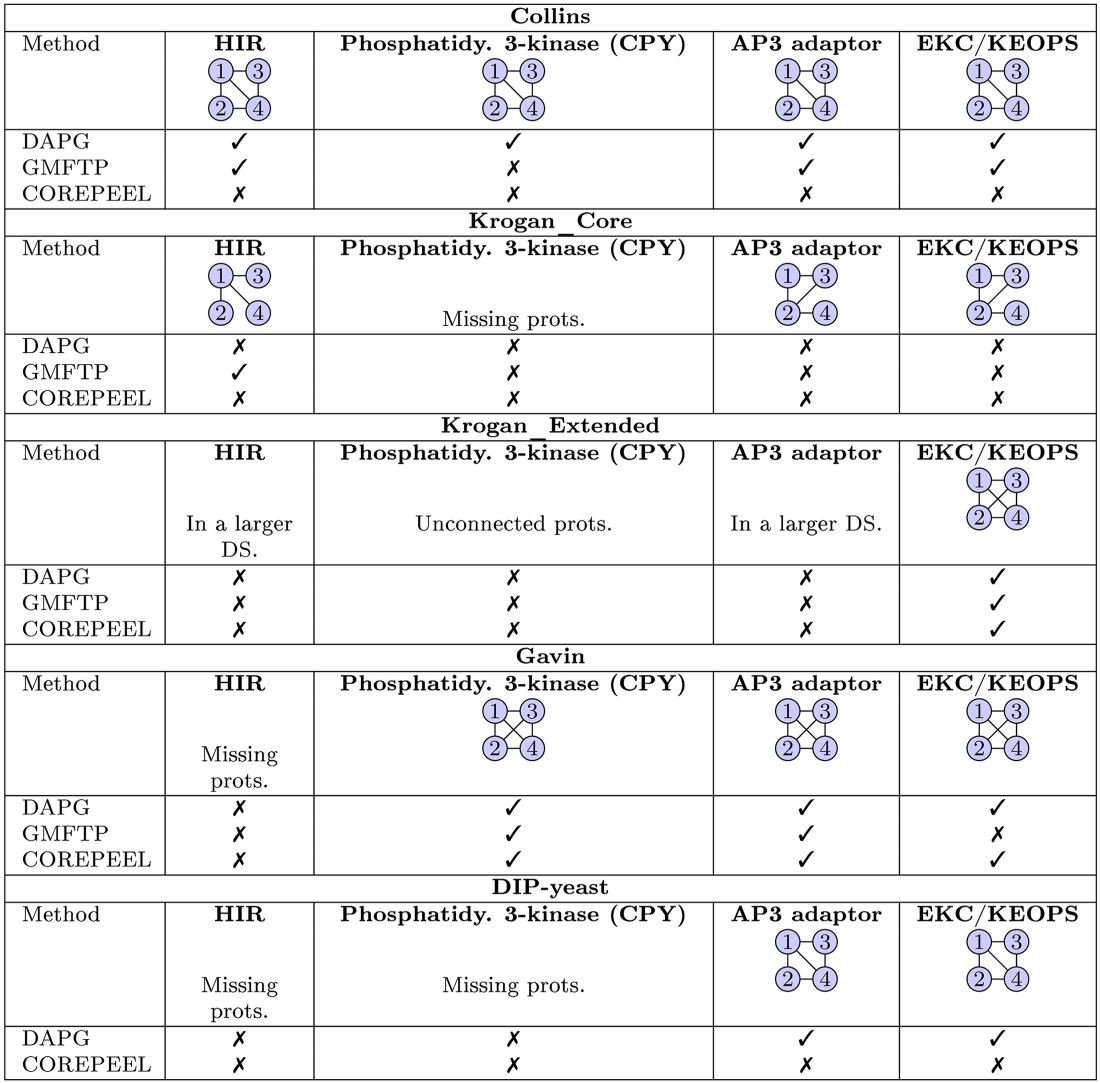
Comparison detection results for a small dense subgraph pattern.

Fig 4 shows the results, where we include the graph pattern in which the complex is present in each PPI. We mark each complex with a ✔ mark if the method is able to detect the protein complex with *OS* > = 0.8 and with a ✘ mark otherwise.

Besides, we found two more complexes in DIP-yeast that follow the same pattern, i.e., a clique of four proteins missing an edge in the PPI. In both cases DAPG detects them, but COREPEEL does not. These complexes are:

- alpha, alpha-trehalose-phosphate synthase complex: TPS1, TPS3, TPS2, TSL1
- STE5-MAPK complex: FUS3, STE5, STE7, STE11

Finally, we performed a comparison based on the ability of the method to detect protein complexes with proteins that participate in more than one complex. We considered complexes in the CYC2008 gold standard. Table 17 shows how well each method detects these protein complexes.

**Table 17:** Performance comparison results based on Overlap Score (OS) in detecting overlapping complexes in Collins with gold standard CYC2008.

Collins				
Protein	Complex	DAPG OS	GMFTP OS	COREPEEL OS
TAF14	Ino80p	1.000	0.758	1.000
	TFIIF	0.231	0.750	-
	NuA3	-	-	-
	SWI/SNF	-	-	-
	TFIID	-	-	-
SWD2	Compass	1.000	1.000	0.875
	mRNA cleavage and polyadenylation	0.933	0.871	0.871
ARP4, ACT1	NuA4	0.923	0.923	0.923
	Swr1p	0.852	-	0.769
	Ino80p	-	-	-
NGG1	SAGA	0.789	0.895	0.895
	SLIK	0.663	-	0.420
	Ada2p	0.267	-	-
TAF5, TAF6, TAF9, TAF10	SAGA	0.789	0.895	0.895
	SLIK	0.663	-	0.420
	TFIID	0.667	0.733	0.667
ARP7, ARP9	RSC	1.000	1.000	1.000
	SWI/SNF	0.833	0.833	0.750

### False positive analysis

Predicting protein complexes is challenging because PPI networks are noisy and incomplete, and references are also incomplete and not systematically updated. All prediction techniques report false positives (i.e., predicted complexes that are not in references), although they can be real complexes not included in references or not discovered yet. In this work, we perform an automatic false positive evaluation of predicted complexes for yeast and human that are absent in available references. Our goal is to see if the reported false positives contain interesting gene sets. In this work, we analyze the reported false positives by looking into curated biological databases such as PDBe (Protein Database Bank in Europe, http://www.ebi.ac.uk/pdbe) which contain information about protein complexes that have purified and structurally characterized. Most of the protein complexes in PDBe are small and are absent in gold standards such as CYC2008 and CORUM, mainly because these gold standards have not been updated recently. In addition, PDBe does not have directly available a repository of all the protein complexes it contains. Therefore, here we propose an automated procedure to query the database to find out whether sets of genes are registered as purified complexes in PDBe. Our analysis do not include protein complexes already found in gold standards (i.e., CY2008, SGD, and MIPS for yeast, and CORUM and PCDq for human). In addition, we also include information of protein complexes that have been topologically characterized, a study done by Ahnert et al. [30] and available in the periodic table of protein complexes (http://www.periodicproteincomplexes.org). However, this periodic table is not up to date. In order to automate the procedure we use the following PDBe related databases.

- Uniprot (http://www.uniprot.org). To obtain protein ids related to pdb ids.
- EMBL-EBI Sifts (https://www.ebi.ac.uk/pdbe/docs/sifts/quick.html). To get chain information of proteins.
- PDBe REST API (http://www.ebi.ac.uk/pdbe/pdbe-rest-api). To query for specific PDB id entry summary information (structure, name, title, release dates).
- Protein Complex Periodic Table (http://www.periodicproteincomplexes.org). To query and visualize topology information of heteromeric complexes.

The false positive automatic analysis can be summarized in the following steps.

1. Obtain the yeast and human database including PDB ids from *Uniprot database*, and the *Sifts database*, which contain the protein domains or chains associated with proteins.
2. For each false positive complex, we find the pdb ids for each protein with corresponding chains.
  - We define a potential protein complex if the complex contains at least two proteins that share the same pdb id.
  - We discard a potential protein complex if the complex is part of a protein complex in a gold standard.
3. Look up the pdb ids of potential protein complexes using PDBe REST API database and checking whether it is a heteromeric complex or not based on the entry summary information.
4. Look up the potential protein complex in the Complex Periodic Table and obtaining its information about of subunits and number of repeats as well as its topology. It is important to note that it might be a variation in the number of subunits and repeats with respect to the information on PDBe. This variation might be because the periodic table is not up to date.

Tables 18 and 19 display a subset of candidate protein complexes in PDBe for yeast and human that are not in any gold standard and are present in the Periodic Table of Protein Complexes. The complete list of candidate protein complexes we detected for both organisms is available in the software distribution (files with extension .csv).

**Table 18:** Predicted complexes in Yeast not present in CYC2008, SGD, and MIPS references. Column with Gene ids contains the genes we found in a complex (number of gene ids).

Pdb id	Form name	Gene ids	PDBe Title	url	Periodic Table
2cg9	hetero tetramer	HSP82 SBA1 (2/2)	CRYSTAL STRUCTURE OF AN HSP90-SBA1 CLOSED CHAPERONE COMPLEX (release date: 20060412)	http://www.ebi.ac.uk/pdbe/entry/pdb/2cg9	2 subunits, 2 repeats
3rui	hetero tetramer	ATG7 ATG8 (2/2)	Crystal structure of Atg7C-Atg8 complex (release date: 20111123)	http://www.ebi.ac.uk/pdbe/entry/pdb/3rui	2 subunits, 2 repeats
2z5c	hetero trimer	IRC25 POC4 (2/3)	Crystal Structure of a Novel Chaperone Complex for Yeast 20S Proteasome Assembly (release date: 20080122)	http://www.ebi.ac.uk/pdbe/entry/pdb/2z5c	3 subunits, 1 repeat
3m1i	hetero trimer	CRM1 GSP1 YRB1 (3/3)	Crystal structure of yeast CRM1 (Xpo1p) in complex with yeast RanBP1 (Yrb1p) and yeast RanGTP (Gsp1pGTP) (release date: 20100602)	http://www.ebi.ac.uk/pdbe/entry/pdb/3m1i	3 subunits, 1 repeat
2r25	hetero dimer	SLN1 YPD1 (2/2)	Complex of YPD1 and SLN1-R1 with bound Mg2+ and BeF3- (release date: 20080115)	http://www.ebi.ac.uk/pdbe/entry/pdb/2r25	2 subunits, 1 repeat
2v6x	hetero dimer	DID4 VPS4 (2/2)	STRACTURAL INSIGHT INTO THE INTERACTION BETWEEN ESCRT-III AND VPS4 (release date: 20071016)	http://www.ebi.ac.uk/pdbe/entry/pdb/2v6x	2 subunits, 1 repeat
2z5b	hetero dimer	IRC25 POC4 (2/2)	Crystal Structure of a Novel Chaperone Complex for Yeast 20S Proteasome Assembly (release date: 20080122)	http://www.ebi.ac.uk/pdbe/entry/pdb/2z5b	2 subunits, 1 repeat
3cmm	hetero dimer	UBA1 UBI4 (2/2)	Crystal Structure of the Uba1-Ubiquitin Complex (release date: 20080805)	http://www.ebi.ac.uk/pdbe/entry/pdb/3cmm	2 subunits, 1 repeat
3qml	hetero dimer	KAR2 SIL1 (2/2)	The structural analysis of Sil1-Bip complex reveals the mechanism for Sil1 to function as a novel nucleotide exchange factor (release date: 20110629)	http://www.ebi.ac.uk/pdbe/entry/pdb/3qml	2 subunits, 1 repeat

**Table 19:** Predicted complexes in Human not present in CORUM and PCDq references.

Pdb id	Form name	Gene ids	PDBe Title	url	Periodic Table
4aj5	hetero 30-mer	SKA1 SKA2 SKA3 (3/3)	Crystal structure of the Ska core complex (release date: 20120523)	http://www.ebi.ac.uk/pdbe/entry/pdb/4aj5	3 subunits, 10 repeats
1zgl	hetero 20-mer	HLA-DRA HLA-DRB5 (2/5)	Crystal structure of 3A6 TCR bound to MBP/HLA-DR2a (release date: 20051018)	http://www.ebi.ac.uk/pdbe/entry/pdb/1zgl	4 subunits, 4 repeats
2io3	hetero 12-mer	SENP2 SUMO2 (2/3)	Crystal structure of human Senp2 in complex with RanGAP1-SUMO-2 (release date: 20061114)	http://www.ebi.ac.uk/pdbe/entry/pdb/2io3	3 subunits, 4 repeats
1d0g	hetero hexamer	TNFRSF10B TNFSF10 (2/2)	CRYSTAL STRUCTURE OF DEATH RECEPTOR 5 (DR5) BOUND TO APO2L/TRAIL (release date: 19991022)	http://www.ebi.ac.uk/pdbe/entry/pdb/1d0g	2 subunits, 3 repeats
3l4g	hetero tetramer	FARSA FARSB (2/2)	Crystal structure of Homo Sapiens cytoplasmic Phenylalanyl-tRNA synthetase (release date: 20100309)	http://www.ebi.ac.uk/pdbe/entry/pdb/3l4g	2 subunits, 2 repeats
1hcf	hetero tetramer	NTF4 NTRK2 (2/2)	CRYSTAL STRUCTURE OF TRKB-D5 BOUND TO NEUROTROPHIN-4/5 (release date: 20011206)	http://www.ebi.ac.uk/pdbe/entry/pdb/1hcf	2 subunits, 2 repeats
4dxr	hetero hexamer	SUN2 SYNE1 (2/2)	Human SUN2-KASH1 complex (release date: 20120606)	http://www.ebi.ac.uk/pdbe/entry/pdb/4dxr	1 subunit, 3 repeats
3oj4	hetero trimer	TNFAIP3 UBC UBE2D1 (3/3)	Crystal structure of the A20 ZnF4 (release date: 20101208)	http://www.ebi.ac.uk/pdbe/entry/pdb/3oj4	3 subunits, 1 repeat
1kmc	hetero tetramer	CASP7 XIAP (2/2)	Crystal Structure of the Caspase-7 / XIAP-BIR2 Complex (release date: 20020116)	http://www.ebi.ac.uk/pdbe/entry/pdb/1kmc	1 subunit, 2 repeats
2ibi	hetero dimer	UBC USP2 (2/2)	Covalent Ubiquitin-USP2 Complex (release date: 20061024)	http://www.ebi.ac.uk/pdbe/entry/pdb/2ibi	2 subunits, 1 repeat

## Discussion and conclusions

We have introduced a novel scheme for detecting protein complexes. Our approach is based on modeling PPI networks as directed acyclic graphs, which allowed us to design an efficient mining heuristic for detecting overlapping dense subgraphs considering weighted and unweighted PPI networks. We define protein complexes based on dense subgraphs that usually overlap. An important advantage of our approach is that it enables the easy extension of new traveler and objective functions. New traveler functions might improve the mining process for discovering dense subgraghs and new objective functions might include biological knowledge to discover subgraphs with biological significance. Therefore, further extensions to our framework are based on adding biological information that might improve the discovery of protein complexes or other protein relationships of biological relevance.

We compare our results with state-of-the-art techniques and show that we provide good performance in terms of clustering using different gold standards and biological metrics, as well as good execution times. We show that our method is able to achieve very good results in terms of matching perfectly (*OS* = 1.0) protein complexes in the gold standards. We also provide a post-processing analysis to study false positive complexes that contain proteins in PPI networks that are absent in the gold standards. In order to study false positives, we consider the information available on protein complexes that have been purified and structurally characterized in PDBe. We used this information together with a recent approach that proposes a periodic table for protein complexes that studies different topologies according to the subunits that compose protein complexes. In this study we discovered that more than 50 yeast complexes and more than 300 of false positive human complexes, not present in gold standards, have actually been already characterized and their information is available in PDBe. Many of these complexes have also been found as having an associated type in the periodic table of protein complexes [30]. We propose these “new” real complexes discovered by our approach and already present in such structural databases, to be considered as new candidates for inclusion in the gold standards of protein complexes. Considering these results, we present our list of predicted false-positive protein complexes to the scientific community, conjecturing that at least part of them could be, in fact, true real complexes awaiting to be studied and characterized.

## Supporting information

S1 File Table A1: Mining algorithm. Discovering DSGs in DAPG. Table A2: Detection of an DSG starting at a given node in DAPG. Table A3: Algorithms for redundancy-filtering. Table A4-A17: DAPG results with different parameters and input PPI networks. Table A18-A29: Other method results with different parameters and input PPI networks.
(PDF)
