# Supplementary material for: Protein complex prediction via dense subgraphs and false positive analysis
Source: PLoS One. 2017 Sep 22;12(9):e0183460. doi: 10.1371/journal.pone.0183460 (PMC5609739; doi:10.1371/journal.pone.0183460)
Supplement: S1 File — Table A1: Mining algorithm. Discovering DSGs in DAPG. Table A2: Detection of an DSG starting at a given node in DAPG. Table A3: Algorithms for redundancy-filtering. Table A4-A17: DAPG results with different parameters and input PPI networks. Table A18-A29: Other method results with different parameters and input PPI networks. (PDF) [file pone.0183460.s001.pdf]

Protein Complex Prediction via Dense Subgraphs and False Positive Analysis

C. Hernández, C. Mella, G. Navarro, A. Olivera-Nappa, and J. Araya

September 1, 2017

Contents

1 Supplementary Text **1**

1.1 Algorithm pseudocodes . . . 1

1.1.1 Mining algorithms . . . 1

1.1.2 Protein complex prediction . . . 3

1.2 Weighted and unweighted DAPG . . . 3

1.2.1 Unified DAPG . . . 3

1.3 Methods used for comparison . . . 7

1.3.1 ClusterONE . . . 7

1.3.2 MCL . . . 7

1.3.3 CFinder . . . 7

1.3.4 GMFTP . . . 9

1.3.5 DCAFP . . . 11

1.3.6 RNSC . . . 11

1.3.7 MCODE . . . 12

1.3.8 SPICI . . . 12

1.3.9 COREPEEL . . . 13

2 False positive predicted protein complexes **14**

1 Supplementary Text

1.1 Algorithm pseudocodes

1.1.1 Mining algorithms

Table A1 describes the main mining heuristic, which is based on finding at most one dense subgraph starting at each node in DAPG.

The core of our mining technique is Table A2. It starts at each node v in DAPG and walks its way to the previous node in the path up to a root. Along the path, we maintain in set S the intersection of the $vertexSet$ of the nodes in a subset of the visited nodes (those which provide a better partial DSG), while we maintain in set C the *labels* of the nodes of the selected subset. Note that, at each point, $(S \cup C, S \times C)$ is indeed a valid graph. From all those DSGs, we retain only the "best one". We determine the "best DSG" using and objective function (f_{obj}), which is a configuration parameter.

Table A1: Mining algorithm. Discovering DSGs in DAPG

Require: DAPG representation.
Ensure: list of maximal DSGs (at most $|N(DAPG)|$)
function GetDenseSubGraphs($DAPG$)
$DSGs \leftarrow \emptyset$
for all $node \in V(DAPG)$ **do**
  $nodeDsg \leftarrow$ GetDenseSubgraphFrom($DAPG, node$)
  if $nodeDsg$ is maximal w.r.t. $DSGs$ **then**
    $DSGs \leftarrow DSGs - \{dsg \in DSGs,\ dsg \subset nodeDsg\}$
    $DSGs \leftarrow DSGs \cup \{nodeDsg\}$
  end if
end for
return $DSGs$

Table A2: Detection of an DSG starting at a given node in DAPG.

Require: $node \in N(DAPG)$, $DAPG$, and f_{obj}
Ensure: $bestDSG = (S, C)$, with $node \in C$
function GetDenseSubGraphFrom($DAPG, node$)
$bestDSG \leftarrow (node.vertexSet, \{node.label\})$
$nextNode \leftarrow$ getTravelerNextNode($DAPG, node$)
while $nextNode \neq NULL$ **do**
  $candidate.C \leftarrow bestDSG.C \cup nextNode.label$
  $candidate.S \leftarrow bestDSG.S \cap nextNode.vertexSet$
  if $f_{obj}(bestDSG) < f_{obj}(candidate)$ **then**
    $bestDSG \leftarrow candidate$
  end if
  $nextNode \leftarrow$ getTravelerNextNode($DAPG, nextNode$)
end while
return $bestDSG$

Table A3: Algorithms for redundancy-filtering.

Require: $candidatesSet, threshold, filter$
Ensure: $CC \subseteq candidatesSet$
$CC \leftarrow \emptyset$
for $candidate \in candidatesSet$ **do**
    $AddToFilteredSet(CC, candidate, threshold, filter)$
end for
return CC

Require: $CC, candidate, threshold, filter$
$B \leftarrow cc \in CC : OS(cc, candidate)$ is maximum
if $filter = NONE\ or\ OS(B, candidate) < threshold$ **then**
    $CC \leftarrow CC \cup \{candidate\}$
else
    if $filter = UNION$ **then**
        $CC \leftarrow CC - \{B\}$
        $CC \leftarrow CC \cup \{candidate \cup B\}$
    end if
end if

1.1.2 Protein complex prediction

Table A3 show the way we generate predicted complexes from candidate complexes based on two different filter options: NONE, where a predicted complex is always a candidate complex, and UNION, where a predicted complex is formed by the set union of the complex pairs with overlap score greater than a *threshold* (we used $threshold = 0.8$).

1.2 Weighted and unweighted DAPG

In this section we present all results we obtained in terms of clustering metrics using the unified version of DAPG on weighted and unweighted PPI networks. Therefore here we present the results using different orders and merge options and objective functions for the mining algorithm explained in the main manuscript. Tables A4, A6, A8, A10 show the results using $f_{obj} = |S \cap C|$ and Tables A5, A7, A9, A11 show the results using f_{obj} based on weighted density definitions on small yeast PPI networks. Similarly we present the results on large PPI networks for yeast and human in Tables A12, A14, A16 with $f_{obj} = |S \cap C|$ and Tables A13, A15, A17 with $f_{obj} = WDEGREE$ for measuring the performance using weighter density metrics.

In all experiments we used yeast PPI networks and reference CYC2008 [1] provided by clusterONE software distribution for yeast and PCDq for human. All experiments were performed with total order function ϕ

1.2.1 Unified DAPG

Table A4: Results of clustering using DAPGUU varying algorithm parameters in Collins with complexes of minimum size 3.

order	merge	Complexes	FMeasure	Acc	MMR	Ex. time(s)
ID	NONE	537	0.7203	0.7188	0.6837	1.15
ID	UNION	447	0.6782	0.7115	0.6749	1.06
FREQ	NONE	611	0.7245	0.7241	0.7014	3.41
FREQ	UNION	479	0.6765	0.7175	0.6849	3.19

Table A5: Results of clustering using PPI weight density DAPGUW varying algorithm parameters in Collins with complexes of minimum size 3.

density	order	merge	Complexes	FMeasure	Acc	MMR	Ex. time(s)
WDEGR	ID	NONE	484	0.7013	0.6283	0.4024	2.47
WDEGR	ID	UNION	421	0.7109	0.7006	0.6396	2.38
WDEGR	FREQ	NONE	530	0.7253	0.6485	0.4291	4.48
WDEGR	FREQ	UNION	447	0.7275	0.7065	0.6606	4.40
WEDGE	ID	NONE	363	0.7583	0.6491	0.4532	1.50
WEDGE	ID	UNION	346	0.7825	0.6991	0.6442	1.44
WEDGE	FREQ	NONE	397	0.7807	0.6541	0.4588	3.79
WEDGE	FREQ	UNION	347	0.7852	0.6891	0.6312	3.63
FWDEG	ID	NONE	416	0.7172	0.6291	0.3962	2.80
FWDEG	ID	UNION	334	0.7130	0.6700	0.5324	2.64
FWDEG	FREQ	NONE	424	0.7273	0.6536	0.4035	4.32
FWDEG	FREQ	UNION	320	0.7031	0.6666	0.4950	4.12
FWEDG	ID	NONE	319	0.6865	0.6103	0.3721	1.51
FWEDG	ID	UNION	328	0.7189	0.6506	0.5332	1.50
FWEDG	FREQ	NONE	359	0.7275	0.6280	0.3525	3.60
FWEDG	FREQ	UNION	328	0.7376	0.6548	0.4694	3.53

Table A6: Results of clustering DAPGUU varying algorithm parameters in Krogan Core with complexes of minimum size 3.

order	merge	Complexes	FMeasure	Acc	MMR	Ex. time(s)
ID	NONE	584	0.6376	0.6470	0.4742	1.40
ID	UNION	557	0.6198	0.6422	0.4807	1.32
FREQ	NONE	627	0.6379	0.6116	0.4550	6.23
FREQ	UNION	582	0.5996	0.6137	0.4591	6.16

Table A7: Results of clustering using PPI weight density DAPGUW varying algorithm parameters in Krogan Core with complexes of minimum size 3.

density	order	merge	Complexes	FMeasure	Acc	MMR	Ex. time(s)
WDEGR	ID	NONE	706	0.6404	0.6305	0.4079	2.57
WDEGR	ID	UNION	680	0.6136	0.6506	0.4838	2.48
WDEGR	FREQ	NONE	756	0.6116	0.5992	0.4016	6.71
WDEGR	FREQ	UNION	733	0.5923	0.6119	0.4555	6.75
WEDGE	ID	NONE	441	0.6568	0.6206	0.3744	1.93
WEDGE	ID	UNION	447	0.6558	0.6445	0.4425	1.89
WEDGE	FREQ	NONE	506	0.6436	0.5920	0.3878	6.21
WEDGE	FREQ	UNION	486	0.6242	0.6015	0.4318	6.22
FWDEG	ID	NONE	545	0.6519	0.6389	0.3782	2.31
FWDEG	ID	UNION	521	0.6259	0.6625	0.4403	2.23
FWDEG	FREQ	NONE	575	0.6273	0.6038	0.3700	6.44
FWDEG	FREQ	UNION	549	0.6124	0.6146	0.4121	6.41
FWEDG	ID	NONE	392	0.6428	0.6058	0.3509	1.93
FWEDG	ID	UNION	391	0.6379	0.6265	0.4177	1.95
FWEDG	FREQ	NONE	481	0.6463	0.5653	0.3439	6.29
FWEDG	FREQ	UNION	467	0.6358	0.5785	0.3900	6.24

Table A8: Results of clustering using DAPGUU varying algorithm parameters in Krogan Extended with complexes of minimum size 3.

order	merge	Complexes	FMeasure	Acc	MMR	Ex. time(s)
ID	NONE	896	0.5121	0.6147	0.4352	3.40
ID	UNION	865	0.4847	0.6247	0.4474	3.26
FREQ	NONE	922	0.5027	0.6057	0.4058	15.07
FREQ	UNION	906	0.4763	0.6129	0.4166	15.29

Table A9: Results of clustering using PPI weight density DAPGUW varying algorithm parameters in Krogan Extended with complexes of minimum size 3.

density	order	merge	Complexes	FMeasure	Acc	MMR	Ex. time(s)
WDEGR	ID	NONE	1174	0.5503	0.5999	0.4004	8.31
WDEGR	ID	UNION	1118	0.5175	0.6144	0.4581	8.08
WDEGR	FREQ	NONE	1209	0.5469	0.5809	0.4096	17.94
WDEGR	FREQ	UNION	1150	0.5244	0.5831	0.4176	17.46
WEDGE	ID	NONE	529	0.6131	0.5778	0.3637	5.80
WEDGE	ID	UNION	532	0.5952	0.5982	0.4039	5.85
WEDGE	FREQ	NONE	573	0.6039	0.5705	0.3725	15.70
WEDGE	FREQ	UNION	564	0.5850	0.5765	0.3867	15.84
FWDEG	ID	NONE	963	0.6041	0.6130	0.3987	8.68
FWDEG	ID	UNION	906	0.5719	0.6199	0.4484	8.30
FWDEG	FREQ	NONE	1027	0.6168	0.6086	0.4083	17.49
FWDEG	FREQ	UNION	950	0.5763	0.6078	0.4220	17.38
FWEDG	ID	NONE	518	0.6204	0.5837	0.3580	7.19
FWEDG	ID	UNION	522	0.6026	0.5980	0.4049	7.02
FWEDG	FREQ	NONE	592	0.6066	0.5768	0.4018	16.37
FWEDG	FREQ	UNION	590	0.5928	0.5839	0.4121	16.69

Table A10: Results of clustering using DAPGUU varying algorithm parameters in Gavin with complexes of minimum size 3.

order	merge	Complexes	FMeasure	Acc	MMR	Ex. time(s)
ID	NONE	718	0.6042	0.6995	0.5557	1.44
ID	UNION	641	0.5752	0.7055	0.5838	1.34
FREQ	NONE	592	0.6519	0.7039	0.5627	3.39
FREQ	UNION	529	0.6097	0.6928	0.5561	3.29

Table A11: Results of clustering using PPI weight density DAPGUW varying algorithm parameters in Gavin with complexes of minimum size 3.

density	order	merge	Complexes	FMeasure	Acc	MMR	Ex. time(s)
WDEGR	ID	NONE	699	0.6181	0.6624	0.4300	2.61
WDEGR	ID	UNION	657	0.5814	0.7039	0.5791	2.51
WDEGR	FREQ	NONE	618	0.6489	0.6477	0.4294	3.80
WDEGR	FREQ	UNION	581	0.6165	0.6704	0.5632	3.77
WEDGE	ID	NONE	511	0.6746	0.6600	0.4064	1.74
WEDGE	ID	UNION	489	0.6412	0.6903	0.5311	1.70
WEDGE	FREQ	NONE	465	0.6389	0.6529	0.4054	3.44
WEDGE	FREQ	UNION	460	0.6250	0.6879	0.5243	3.40
FWDEG	ID	NONE	530	0.6705	0.6751	0.4218	2.25
FWDEG	ID	UNION	482	0.6081	0.6774	0.4740	2.23
FWDEG	FREQ	NONE	384	0.6766	0.6407	0.3247	3.71
FWDEG	FREQ	UNION	347	0.6175	0.6330	0.3779	3.60
FWEDG	ID	NONE	436	0.6239	0.6387	0.3842	1.78
FWEDG	ID	UNION	461	0.6076	0.6702	0.4735	1.77
FWEDG	FREQ	NONE	425	0.5889	0.6046	0.3224	3.98
FWEDG	FREQ	UNION	426	0.5740	0.6326	0.4031	3.51

Table A12: Results of clustering using DAPGUU varying algorithm parameters in Biogrid yeast with complexes of minimum size 3.

order	merge	Complexes	FMeasure	Acc	MMR	Ex. time(s)
ID	NONE	4,992	0.1490	0.5692	0.3385	345.77
ID	UNION	4,945	0.1444	0.5693	0.3371	346.42
FREQ	NONE	5,012	0.1528	0.5663	0.3455	757.74
FREQ	UNION	4,980	0.1486	0.5702	0.3523	751.00

Table A13: Results of clustering using DAPGUWD varying algorithm parameters in Biogrid yeast with complexes of minimum size 3.

density	order	merge	Complexes	FMeasure	Acc	MMR	Ex. time(s)
WDEGR	ID	NONE	5,199	0.0620	0.4000	0.1506	798.1
WDEGR	ID	UNION	5,153	0.0600	0.4016	0.1521	806.2
WDEGR	FREQ	NONE	4,803	0.0331	0.3514	0.0769	1,315.1
WDEGR	FREQ	UNION	4,747	0.0323	0.3519	0.0771	1,324.6

Table A14: Results of clustering using DAPGUU varying algorithm parameters in HPRD with complexes of minimum size 3 and reference PCDq.

order	merge	Complexes	FMeasure	Acc	MMR	Ex. time(s)
ID	NONE	2,440	0.3292	0.3122	0.1519	62.04
ID	UNION	2,442	0.3266	0.3134	0.1544	58.70
FREQ	NONE	2,368	0.3212	0.2835	0.1323	229.3
FREQ	UNION	2,400	0.3241	0.2884	0.1394	259.5

1.3 Methods used for comparison

In order to evaluate DAPG in detecting protein complexes, we used the following state-of-the-art methods: ClusterONE [2], MCL [3], Cfinder [4], and GMFTP [5]. The performance of each method depends on its parameter setting and the reference (gold standard) of protein complexes used as ground truth. Therefore, we first describe the main features of each of algorithms and provide the parameter tuning using the reference CYC2008 [1]. We optimized the parameters that achieved the best results based on MMR (Maximum Matching Ratio), proposed by clusterONE, and used the implementation for measuring clustering metrics provided by them and available at `http://www.paccanarolab.org/static_content/clusterone/additional_information.html`. All experiments report the parameters and the clustering metrics: FMeasure, Acc, MMR as well as the execution time in seconds.

1.3.1 ClusterONE

ClusterONE detects overlapping protein complexes from weighted and unweighted PPI networks, and it is based on overlapping neighborhood expansion. The main parameter of clusterONE is d, which is the minimum density of clusters, and we keep the other parameters as given by default as has been used in previous work [5]. Tables A18, A19, A20, A21, and A24 present the results for Collins, KroganCore, KroganExt, Gavin and Biogrid.

1.3.2 MCL

MCL is based on detecting clusters using a model that uses random walks on the input graph adopting Markov Chains trying to discover where the flows concentrate forming clusters. The Inflation (I) parameter is its key parameter, which tunes the granularity of the clusters. We executed MCL using different inflations for all input PPI networks and we provide the results in Tables A25, A26, A27, A28 and A29.

1.3.3 CFinder

CFinder is based on Clique Percolation Method (CPM) [6] to detect overlapping modules in biological networks. The CPM method consists of building communities from k-cliques where a community is defined as the maximal union of k-cliques that are connected through a series of adjacent cliques. The keys parameters in CFinder are the parameter k (for size k in k-clique) and the parameter t which represents the time in seconds allowed for searching a clique

Table A15: Results of clustering using DAPGUWD varying algorithm parameters in HPRD with complexes of minimum size 3 and reference PCDq.

density	order	merge	Complexes	FMeasure	Acc	MMR	Ex. time(s)
WDEGR	ID	NONE	3,791	0.3390	0.2821	0.1790	121.2
WDEGR	ID	UNION	3,751	0.3346	0.2869	0.1832	120.5
WDEGR	FREQ	NONE	3,592	0.3190	0.2588	0.1506	287.9
WDEGR	FREQ	UNION	3,562	0.3216	0.2631	0.1603	291.7

Table A16: Results of clustering using DAPGUU varying algorithm parameters in Biogrid human with complexes of minimum size 3.

order	merge	Complexes	FMeasure	Acc	MMR	Ex. time(s)
ID	NONE	7,178	0.1496	0.3534	0.1247	900.0
ID	UNION	7,105	0.1469	0.3529	0.1249	900.2
FREQ	NONE	7,360	0.1577	0.3470	0.1303	3,238.2
FREQ	UNION	7,314	0.1542	0.3485	0.1319	3,210.3

Table A17: Results of clustering using DAPGUWD varying algorithm parameters in Biogrid human with complexes of minimum size 3.

density	order	merge	Complexes	FMeasure	Acc	MMR	Ex. time(s)
WDEGR	ID	NONE	11,160	0.0932	0.2516	0.0951	1,803.2
WDEGR	ID	UNION	10,871	0.0978	0.2529	0.0964	1,795.3
WDEGR	FREQ	NONE	11,233	0.1037	0.2386	0.1028	3,013.2
WDEGR	FREQ	UNION	10,727	0.0986	0.2407	0.1009	3,012.4

Table A18: Results of clustering using ClusterONE varying algorithm parameters in Collins with complexes of minimum size 3.

D	Complexes	FMeasure	Acc	MMR	Ex. time(s)
0.1	172	0.6936	0.7448	0.5626	1.35
0.2	177	0.6844	0.7469	0.5656	1.34
0.3	194	0.6646	0.7343	0.5707	1.39
0.4	187	0.6940	0.7677	0.5711	1.37
0.5	169	0.7273	0.7676	0.5477	1.33
0.6	136	0.7594	0.7376	0.5132	1.30
0.7	128	0.7836	0.7208	0.5061	1.28
0.8	117	0.7615	0.6816	0.4559	1.24
0.9	100	0.6996	0.6564	0.3861	1.19

from a node in the graph. For all PPI networks we found that our best results we for $k = 3$ for Collins, and KroganCore, $k = 4$ for KroganExtended and Gavin, and $K = 6$ for Biogrid. For the t parameters we found that using $t = 1$ or $t = 10$ in Collins, KroganCore, KroganExtended and Gavin all results were the same so we reported execution times for $t = 1$. However, in the case of Biogrid the execution time with $t = 10$ took more than 2 days so we used $t = 1$.

Table A19: Results of clustering using ClusterONE varying algorithm parameters in Krogan Core with complexes of minimum size 3.

D	Complexes	FMeasure	Acc	MMR	Ex. time(s)
0.1	372	0.4852	0.7494	0.4424	1.80
0.2	456	0.5099	0.7674	0.4600	1.79
0.3	522	0.4637	0.7683	0.4817	1.68
0.4	411	0.5844	0.7416	0.5068	1.65
0.5	240	0.6836	0.7075	0.4598	1.63
0.6	183	0.7128	0.6622	0.4055	1.56
0.7	139	0.6729	0.6203	0.3598	1.44
0.8	111	0.5903	0.5637	0.2753	1.39
0.9	79	0.5649	0.4859	0.2099	1.31

Table A20: Results of clustering using ClusterONE varying algorithm parameters in Krogan Extended with complexes of minimum size 3.

D	Complexes	FMeasure	Acc	MMR	Ex. time(s)
0.1	421	0.4550	0.7145	0.4081	2.34
0.2	504	0.4587	0.7229	0.4197	2.33
0.3	530	0.4693	0.7334	0.4342	2.21
0.4	402	0.5751	0.7050	0.4553	2.18
0.5	257	0.6497	0.6614	0.4188	1.98
0.6	200	0.6923	0.6478	0.3850	1.88
0.7	142	0.6334	0.5915	0.3211	1.84
0.8	112	0.5879	0.5320	0.2505	1.82
0.9	82	0.5512	0.4733	0.1922	1.79

Table A21: Results of clustering using ClusterONE varying algorithm parameters in Gavin with complexes of minimum size 3.

D	Complexes	FMeasure	Acc	MMR	Ex. time(s)
0.1	254	0.5383	0.7548	0.4949	1.45
0.2	239	0.5706	0.7626	0.5102	1.46
0.3	196	0.6852	0.7523	0.5378	1.41
0.4	173	0.7842	0.6953	0.5171	1.43
0.5	153	0.7740	0.6229	0.4214	1.42
0.6	89	0.5868	0.4757	0.2769	1.29
0.7	34	0.3626	0.2991	0.1239	1.16
0.8	5	0.0556	0.1089	0.0126	1.17

1.3.4 GMFTP

GMFTP is based on a generative model with functional and topological properties tending to predict protein complexes that are formed by group of proteins which frequently interact with each other and have similar functional patterns. The method transform the detection problem into a parameter estimation problem. The objective function in GMFTP is not convex and then the multiplicative updating rules of the algorithm does not necessarily converge to the global minimum. As a result, the method cannot guarantee the final estimator is the

Table A22: Results of clustering using ClusterONE varying algorithm parameters in Biogrid yeast with complexes of minimum size 3.

D	Complexes	FMeasure	Acc	MMR	Ex. time(s)
0.1	805	0.1406	0.4930	0.0654	
0.2	972	0.1204	0.5256	0.0686	
0.3	1,088	0.0981	0.5729	0.0771	
0.4	1,340	0.1068	0.6098	0.1042	
0.5	1,523	0.1031	0.5984	0.1295	
0.6	593	0.2500	0.5643	0.1554	
0.7	369	0.3132	0.5426	0.1599	
0.8	332	0.3217	0.5342	0.1553	
0.9	183	0.3206	0.4741	0.1304	

Table A23: Results of clustering using ClusterONE varying algorithm parameters in human HPRD with complexes of minimum size 3.

D	Complexes	FMeasure	Acc	MMR	Ex. time(s)
0.1	1,287	0.2300	0.4804	0.0774	
0.2	1,410	0.2783	0.4920	0.1002	
0.3	1,639	0.3063	0.5051	0.1359	
0.4	1,867	0.3081	0.5033	0.1538	
0.5	2,186	0.2923	0.5122	0.1718	
0.6	786	0.2651	0.4285	0.0968	
0.7	217	0.1672	0.2980	0.0382	
0.8	191	0.1640	0.2840	0.0371	
0.9	75	0.0876	0.2026	0.0195	

Table A24: Results of clustering using ClusterONE varying algorithm parameters in human Biogrid with complexes of minimum size 3.

D	Complexes	FMeasure	Acc	MMR	Ex. time(s)
0.1	2,740	0.0533	0.4227	0.0201	
0.2	2,871	0.0633	0.4493	0.0249	
0.3	3,230	0.0827	0.4658	0.0406	
0.4	3,679	0.0875	0.4701	0.0525	
0.5	4,254	0.0863	0.4802	0.0653	
0.6	977	0.1222	0.3891	0.0376	
0.7	390	0.1195	0.3327	0.0258	
0.8	332	0.1082	0.3108	0.0237	
0.9	170	0.0721	0.2539	0.0166	

globally optimum solution and the result is not deterministic. This issue is addressed by the method having a parameter for repeating the entire calculation, which is the $repeat_times$ parameter. By default this parameter is set to 100.

In our experiments, when trying to execute GMFTP on all PPI networks, we found that using all the default parameters of GMFTP was impossible to get results before a day of execution time. Therefore, we left all parameters as the defaults, except the *repeat_time* which we set to 10 instead of 100. Doing this we were able to get results in a little more than 12 hours of execution with

Table A25: Results of clustering using MCL varying algorithm parameters in Collins with complexes of minimum size 3.

I	Complexes	FMeasure	Acc	MMR	Ex. time(s)
1.2	99	0.5764	0.6562	0.3797	0.74
1.4	130	0.6857	0.7351	0.4604	0.74
1.6	145	0.6899	0.7447	0.4899	0.74
1.8	152	0.6816	0.7435	0.4992	0.74
2.0	155	0.6815	0.7474	0.5121	0.74
2.2	158	0.6813	0.7487	0.5165	0.74
2.4	160	0.6787	0.7465	0.5161	0.74
2.6	165	0.6690	0.7486	0.5215	0.74
2.8	168	0.6690	0.7522	0.5231	0.74
3.0	171	0.6806	0.7870	0.5363	0.74
3.2	173	0.6804	0.7908	0.5452	0.74
3.4	172	0.6897	0.7888	0.5408	0.74
3.6	174	0.6781	0.7841	0.5361	0.74
3.8	176	0.6847	0.7830	0.5366	0.74
4.0	180	0.6756	0.7842	0.5418	0.74
4.2	181	0.6733	0.7845	0.5519	0.74
4.4	181	0.6733	0.7835	0.5510	0.74
4.6	181	0.6800	0.7852	0.5561	0.74
4.8	183	0.6821	0.7814	0.5572	0.74
5.0	184	0.6776	0.7803	0.5591	0.74
5.2	186	0.6797	0.7801	0.5621	0.74
5.4	186	0.6820	0.7803	0.5625	0.74
5.6	185	0.6842	0.7762	0.5614	0.74
5.8	186	0.6820	0.7720	0.5607	0.74
6.0	187	0.6732	0.7693	0.5577	0.74
6.2	187	0.6710	0.7690	0.5581	0.74
6.4	187	0.6731	0.7679	0.5580	0.74
6.6	186	0.6818	0.7680	0.5611	0.74
6.8	188	0.6817	0.7685	0.5675	0.74
7.0	189	0.6837	0.7689	0.5726	0.74
7.2	193	0.6751	0.7681	0.5722	0.74
7.4	196	0.6875	0.7641	0.5760	0.74
7.6	196	0.6916	0.7626	0.5746	0.74
7.8	196	0.6854	0.7627	0.5735	0.74
8.0	196	0.6854	0.7577	0.5707	0.74
8.2	198	0.6935	0.7537	0.5731	0.74
8.4	198	0.6935	0.7533	0.5729	0.74
8.6	201	0.6850	0.7484	0.5712	0.74
8.8	201	0.6869	0.7463	0.5658	0.74
9.0	202	0.6848	0.7447	0.5650	0.74

all PPI networks.

1.3.5 DCAFP

DCAFP is a method that predict protein complexes based on two main properties. The first considers the idea of dense connected proteins in the PPI network and the second is based on the idea that proteins in the same protein complexes are at least similar in specific subsets of funtional GO categories in the context of functional information given in the Gene ontology. DCAFP has three main parameters *minsize attributes*, *delta*, *wmin*, *osmax*, *maxloops*, where the paremeters *wmin* and *osmax* are the more relevants, where *wmin* has more impact in the size of the clusters found and *osmax* is more important in the performance. We modified these parameters between 0.2 and 1.0, keeping the other by default, to obtain our results.

1.3.6 RNSC

RNSC is a stocastic algorithm based on a search meta-heuristic aiming to optimize the network partition to define clusters based on a cost function. The

Table A26: Results of clustering using MCL varying algorithm parameters in Krogan Core with complexes of minimum size 3.

I	Complexes	FMeasure	Acc	MMR	Ex. time(s)
1.2	86	0.2122	0.5220	0.0844	8.62
1.4	203	0.4265	0.7088	0.2604	8.62
1.6	297	0.4282	0.7462	0.3451	8.62
1.8	338	0.4458	0.7620	0.3916	8.62
2.0	367	0.4297	0.7568	0.4072	8.62
2.2	369	0.4225	0.7499	0.4062	8.62
2.4	385	0.4134	0.7378	0.4127	8.62
2.6	393	0.4118	0.7211	0.4077	8.62
2.8	393	0.4073	0.7196	0.4075	8.62
3.0	391	0.4007	0.7135	0.4053	8.62
3.2	392	0.3963	0.7052	0.4022	8.62
3.4	393	0.3905	0.6957	0.3914	8.62
3.6	391	0.3773	0.6869	0.3782	8.62
3.8	386	0.3667	0.6799	0.3660	8.62
4.0	378	0.3684	0.6741	0.3589	8.62
4.2	373	0.3674	0.6687	0.3522	8.62
4.4	372	0.3712	0.6629	0.3509	8.62
4.6	371	0.3719	0.6619	0.3495	8.62
4.8	369	0.3636	0.6496	0.3395	8.62
5.0	366	0.3643	0.6442	0.3313	8.62
5.2	363	0.3664	0.6397	0.3301	8.62
5.4	359	0.3654	0.6359	0.3273	8.62
5.6	358	0.3661	0.6346	0.3269	8.62
5.8	355	0.3643	0.6332	0.3268	8.62
6.0	355	0.3636	0.6290	0.3240	8.62
6.2	354	0.3682	0.6246	0.3237	8.62
6.4	355	0.3668	0.6234	0.3200	8.62
6.6	352	0.3689	0.6202	0.3175	8.62
6.8	346	0.3622	0.6141	0.3054	8.62
7.0	346	0.3590	0.6133	0.3067	8.62
7.2	344	0.3604	0.6163	0.3076	8.62
7.4	344	0.3604	0.6148	0.3071	8.62
7.6	339	0.3655	0.6124	0.3068	8.62
7.8	339	0.3655	0.6108	0.3074	8.62
8.0	337	0.3589	0.6066	0.3005	8.62
8.2	336	0.3556	0.6030	0.2975	8.62
8.4	335	0.3522	0.6005	0.2936	8.62
8.6	333	0.3537	0.5994	0.2922	8.62
8.8	332	0.3455	0.5951	0.2877	8.62
9.0	329	0.3436	0.5950	0.2868	8.62

algorithm has several parameters such as the tabu length, number of experiments, diversification length, and diversification frequency. We run RNSC with default parameters.

1.3.7 MCODE

MCODE is one of the earliest algorithms that provide a solution for protein complex prediction. We use a command line application for linux platform to run the experiments. The method has several parameters, among the most important parameters are the neighborhood density percentage which varies from 0 to 1.0 and the maxdepth parameter, which we set in 1000 and 10000. We defined the other parameters in their default values.

1.3.8 SPICI

SPICI is a method that has a web site to run it and it also has the software available for download at `http://compbio.cs.princeton.edu/spici/` . The method is based on ranking nodes by weighted degree and build clusters greedily

Table A27: Results of clustering using MCL varying algorithm parameters in Krogan Extended with complexes of minimum size 3.

I	Complexes	FMeasure	Acc	MMR	Ex. time(s)
1.2	85	0.1298	0.4373	0.0370	19.50
1.4	232	0.3375	0.6739	0.1871	19.50
1.6	336	0.3386	0.7132	0.2500	19.50
1.8	400	0.3422	0.7337	0.2810	19.50
2.0	436	0.3389	0.7309	0.2895	19.50
2.2	467	0.3333	0.7235	0.2972	19.50
2.4	497	0.3243	0.7168	0.3090	19.50
2.6	517	0.3110	0.7039	0.3079	19.50
2.8	521	0.3059	0.6947	0.3026	19.50
3.0	528	0.3024	0.6921	0.3042	19.50
3.2	531	0.2955	0.6850	0.2999	19.50
3.4	529	0.2882	0.6759	0.2915	19.50
3.6	534	0.2752	0.6674	0.2854	19.50
3.8	531	0.2735	0.6607	0.2785	19.50
4.0	536	0.2663	0.6549	0.2726	19.50
4.2	542	0.2637	0.6443	0.2702	19.50
4.4	538	0.2652	0.6422	0.2690	19.50
4.6	536	0.2595	0.6386	0.2638	19.50
4.8	536	0.2535	0.6341	0.2576	19.50
5.0	535	0.2507	0.6344	0.2557	19.50
5.2	534	0.2511	0.6307	0.2553	19.50
5.4	535	0.2451	0.6261	0.2506	19.50
5.6	532	0.2433	0.6199	0.2470	19.50
5.8	530	0.2411	0.6157	0.2424	19.50
6.0	529	0.2408	0.6091	0.2377	19.50
6.2	528	0.2383	0.6069	0.2359	19.50
6.4	529	0.2323	0.6014	0.2331	19.50
6.6	525	0.2308	0.6015	0.2319	19.50
6.8	523	0.2340	0.5994	0.2291	19.50
7.0	521	0.2346	0.5965	0.2264	19.50
7.2	520	0.2350	0.5959	0.2264	19.50
7.4	518	0.2324	0.5927	0.2252	19.50
7.6	516	0.2277	0.5896	0.2203	19.50
7.8	516	0.2277	0.5883	0.2198	19.50
8.0	513	0.2287	0.5845	0.2182	19.50
8.2	512	0.2261	0.5826	0.2168	19.50
8.4	509	0.2271	0.5804	0.2151	19.50
8.6	510	0.2238	0.5758	0.2096	19.50
8.8	510	0.2264	0.5748	0.2088	19.50
9.0	511	0.2261	0.5725	0.2077	19.50

starting at seed nodes with decreasing degree. Clusters are formed by increasingly adding neighbors of seed vertexes that incrementing their densities. We tried different values for minimum density, including default 0.5 and different values of minimum support threshold. We varied these parameters between 0.2 an 0.8. We also defined the sparcity parameter (-m) with its possible values of 0,1,and 2.

1.3.9 COREPEEL

COREPEEL is a method that predict protein complexes in polynomial running time and works well in large PPI networks. The method is available for running in `http://bioalgo.iit.cnr.it/`. The approach is based on finding dense communities of the form of quasi-cliques. The method has two basic step, the first consist of applying a core decomposition of the graph where for each vertex in a graph provides a tight upper bound to the size of the largest quasi-clique that includes that vertex. And the second step consists of discarding (peeling out) loosely connected vertices from the quasi-cliques. The method has several parameters, such as the minimum density, maxumum size, subgraph min size,

Table A28: Results of clustering using MCL varying algorithm parameters in Gavin with complexes of minimum size 3.

I	Complexes	FMeasure	Acc	MMR	Ex. time(s)
1.2	63	0.3179	0.5173	0.1320	2.01
1.4	154	0.5111	0.7294	0.2984	2.01
1.6	198	0.5190	0.7516	0.3791	2.01
1.8	218	0.5119	0.7555	0.4062	2.01
2.0	224	0.5131	0.7613	0.4268	2.01
2.2	227	0.5187	0.7609	0.4450	2.01
2.4	236	0.5098	0.7600	0.4528	2.01
2.6	236	0.5112	0.7575	0.4558	2.01
2.8	243	0.5193	0.7557	0.4655	2.01
3.0	246	0.5246	0.7443	0.4726	2.01
3.2	253	0.5308	0.7448	0.4794	2.01
3.4	254	0.5372	0.7437	0.4856	2.01
3.6	253	0.5333	0.7331	0.4726	2.01
3.8	253	0.5294	0.7380	0.4801	2.01
4.0	252	0.5201	0.7367	0.4760	2.01
4.2	252	0.5269	0.7361	0.4804	2.01
4.4	252	0.5241	0.7312	0.4782	2.01
4.6	253	0.5227	0.7321	0.4817	2.01
4.8	250	0.5187	0.7286	0.4775	2.01
5.0	250	0.5187	0.7245	0.4770	2.01
5.2	248	0.5147	0.7184	0.4659	2.01
5.4	248	0.5187	0.7130	0.4628	2.01
5.6	244	0.5217	0.7092	0.4552	2.01
5.8	242	0.5232	0.7070	0.4516	2.01
6.0	240	0.5151	0.7049	0.4437	2.01
6.2	240	0.5205	0.7046	0.4451	2.01
6.4	239	0.5220	0.7038	0.4432	2.01
6.6	238	0.5289	0.7025	0.4432	2.01
6.8	238	0.5289	0.7011	0.4443	2.01
7.0	239	0.5275	0.6990	0.4432	2.01
7.2	239	0.5275	0.6972	0.4410	2.01
7.4	240	0.5220	0.6960	0.4399	2.01
7.6	240	0.5301	0.6928	0.4366	2.01
7.8	238	0.5289	0.6947	0.4353	2.01
8.0	237	0.5249	0.6942	0.4341	2.01
8.2	237	0.5193	0.6894	0.4284	2.01
8.4	235	0.5167	0.6889	0.4250	2.01
8.6	236	0.5111	0.6883	0.4250	2.01
8.8	234	0.5125	0.6819	0.4171	2.01
9.0	233	0.5140	0.6823	0.4167	2.01

filter type (strict, medium, loose) and maximum jaccard separation. We tried between 50 and 100 minimum density, maximum jaccard separation between 0.5 and 1.0 and all the three filter types in all PPI networks.

2 False positive predicted protein complexes

Predicted protein complexes considered as false positive, i.e, protein complexes that are absent in gold standards are analyzed based on the information stored in PDBe containing protein complexes that have been characterized structurally. Many of these PDB ids are present in the Periodic table of protein complexes [7]. We report the complete lists for these candidate complexes in files with the extension .csv. Additionally we report predicted protein complexes found to be false positive that include these candidate protein complexes. This information is stored in files with the extension .xml. Both types of files are included in the results directory included in the software distribution developed in our approach (`http://doi.org/10.6084/m9.figshare.5297314.v1`).

Table A29: Results of clustering using MCL varying algorithm parameters in Biogrid with complexes of minimum size 3.

I	Complexes	FMeasure	Acc	MMR	Ex. time(s)
1.6	7	0.0082	0.0436	0.0021	59.13
1.8	35	0.0444	0.1208	0.0140	59.13
2.0	88	0.0807	0.2015	0.0298	59.13
2.2	167	0.1100	0.2976	0.0496	59.13
2.4	224	0.1641	0.3621	0.0891	59.13
2.6	265	0.2195	0.4164	0.1216	59.13
2.8	300	0.2362	0.4620	0.1454	59.13
3.0	315	0.2560	0.4645	0.1510	59.13
3.2	330	0.2648	0.4653	0.1569	59.13
3.4	340	0.2355	0.4695	0.1442	59.13
3.6	347	0.2334	0.4717	0.1507	59.13
3.8	338	0.2372	0.4748	0.1476	59.13
4.0	334	0.2384	0.4651	0.1404	59.13
4.2	326	0.2274	0.4567	0.1325	59.13
4.4	315	0.2308	0.4539	0.1267	59.13
4.6	310	0.2230	0.4603	0.1296	59.13
4.8	306	0.2097	0.4533	0.1213	59.13
5.0	301	0.1966	0.4486	0.1157	59.13
5.2	296	0.1985	0.4479	0.1149	59.13
5.4	292	0.1962	0.4442	0.1103	59.13
5.6	288	0.2054	0.4415	0.1171	59.13
5.8	281	0.2074	0.4361	0.1161	59.13
6.0	275	0.2099	0.4337	0.1159	59.13
6.2	275	0.2059	0.4498	0.1125	59.13
6.4	272	0.2072	0.4438	0.1079	59.13
6.6	271	0.2036	0.4355	0.1048	59.13
6.8	269	0.2004	0.4305	0.1015	59.13
7.0	263	0.1947	0.4287	0.0986	59.13
7.2	258	0.1926	0.4176	0.0959	59.13
7.4	253	0.1860	0.4089	0.0892	59.13
7.6	249	0.1792	0.4020	0.0846	59.13
7.8	246	0.1719	0.3950	0.0814	59.13
8.0	243	0.1688	0.3895	0.0806	59.13
8.2	242	0.1691	0.3866	0.0808	59.13
8.4	241	0.1695	0.3852	0.0809	59.13
8.6	234	0.1591	0.3751	0.0738	59.13
8.8	233	0.1548	0.3729	0.0718	59.13
9.0	231	0.1469	0.3672	0.0678	59.13

References

[1] Pu, S., Wong, J., Turner, B., Cho, E., Wodak, S.J.: Up-to-date catalogues of yeast protein complexes. Nucleic acids research **37**(3), 825–831 (2009)

[2] Nepusz, T., Yu, H., Paccanaro, A.: Detecting overlapping protein complexes in protein-protein interaction networks. Nature methods **9**(5), 471–472 (2012)

[3] Enright, A.J., Van Dongen, S., Ouzounis, C.A.: An efficient algorithm for large-scale detection of protein families. Nucleic acids research **30**(7), 1575–1584 (2002)

[4] Adamcsek, B., Palla, G., Farkas, I.J., Derényi, I., Vicsek, T.: Cfinder: locating cliques and overlapping modules in biological networks. Bioinformatics **22**(8), 1021–1023 (2006)

[5] Zhang, X.-F., Dai, D.-Q., Ou-Yang, L., Yan, H.: Detecting overlapping protein complexes based on a generative model with functional and topological properties. BMC bioinformatics **15**(1), 186 (2014)

[6] Palla, G., Derényi, I., Farkas, I., Vicsek, T.: Uncovering the overlapping community structure of complex networks in nature and society. Nature **435**(7043), 814–818 (2005)

[7] Ahnert, S.E., Marsh, J.A., Hernández, H., Robinson, C.V., Teichmann, S.A.: Principles of assembly reveal a periodic table of protein complexes. Science **350**(6266) (2015). doi:10.1126/science.aaa2245. http://science.sciencemag.org/content/350/6266/aaa2245.full.pdf
